# Metabolic dynamics and prediction of sFGR and adverse fetal outcomes: a prospective longitudinal cohort study

**DOI:** 10.1186/s12916-023-03134-9

**Published:** 2023-11-23

**Authors:** Nana Huang, Wei Chen, Hai Jiang, Jing Yang, Youzhen Zhang, Huifeng Shi, Ying Wang, Pengbo Yuan, Jie Qiao, Yuan Wei, Yangyu Zhao

**Affiliations:** 1 Department of Obstetrics and Gynecology, Peking University Third Hospital, No. 49 Huayuan North Road, Beijing, 100191 China; 2 National Clinical Research Center for Obstetrics and Gynecology (Peking University Third Hospital), Beijing, China; 3 National Center for Healthcare Quality Management in Obstetrics, Beijing, China; 4 Key Laboratory of Assisted Reproduction (Peking University), Ministry of Education, Beijing, China; 5 Beijing Key Laboratory of Reproductive Endocrinology and Assisted Reproductive Technology, Beijing, China; 6 State Key Laboratory of Female Fertility Promotion, Center for Reproductive Medicine, Department of Obstetrics and Gynecology, Peking University Third Hospital, No. 49 Huayuan North Road, Beijing, 100191 China; 7 Beijing Advanced Innovation Center for Genomics, Beijing, China; 8 Peking-Tsinghua Center for Life Sciences, Peking University, Beijing, China

**Keywords:** Selective fetal growth restriction, Twins, Brain injury, Metabolism, Prediction

## Abstract

**Background:**

Selective fetal growth restriction (sFGR) is an extreme complication that significantly increases the risk of perinatal mortality and long-term adverse neurological outcomes in offspring, affecting approximately 15% of monochorionic diamniotic (MCDA) twin pregnancies. The lack of longitudinal cohort studies hinders the early prediction and intervention of sFGR.

**Methods:**

We constructed a prospective longitudinal cohort study of sFGR, and quantified 25 key metabolites in 337 samples from maternal plasma in the first, second, and third trimester and from cord plasma. In particular, our study examined fetal growth and brain injury data from ultrasonography and used the Ages and Stages Questionnaire-third edition subscale (ASQ-3) to evaluate the long-term neurocognitive behavioral development of infants aged 2–3 years. Furthermore, we correlated metabolite levels with ultrasound data, including physical development and brain injury indicators, and ASQ-3 data using Spearman’s-based correlation tests. In addition, special combinations of differential metabolites were used to construct predictive models for the occurrence of sFGR and fetal brain injury.

**Results:**

Our findings revealed various dynamic patterns for these metabolites during pregnancy and a maximum of differential metabolites between sFGR and MCDA in the second trimester (*n* = 8). The combination of l-phenylalanine, l-leucine, and l-isoleucine in the second trimester, which were closely related to fetal growth indicators, was highly predictive of sFGR occurrence (area under the curve [AUC]: 0.878). The combination of l-serine, l-histidine, and l-arginine in the first trimester and creatinine in the second trimester was correlated with long-term neurocognitive behavioral development and showed the capacity to identify fetal brain injury with high accuracy (AUC: 0.94).

**Conclusions:**

The performance of maternal plasma metabolites from the first and second trimester is superior to those from the third trimester and cord plasma in discerning sFGR and fetal brain injury. These metabolites may serve as useful biomarkers for early prediction and promising targets for early intervention in clinical settings.

**Supplementary Information:**

The online version contains supplementary material available at 10.1186/s12916-023-03134-9.

## Background

Monochorionic diamniotic (MCDA) twins, a complex twin pregnancy, often lead to disastrous pregnancy complications due to the existence of shared placenta and anastomotic vessels [1, 2], typically caused by selective fetal growth restriction (sFGR) [3, 4]. In clinical practice, sFGR is defined as an estimated fetal weight < 10th percentile and fetal weight discordance ≥ 20% or 25% [5, 6]. It significantly increases the risk of perinatal mortality and long-term adverse infant neurological outcomes [1], affecting approximately 15% of MCDA twin pregnancies [3, 4]. Currently, the diagnosis and classification of sFGR can only rely on ultrasonography findings in the second or third trimester [7], and fetal brain injury is often assessed after birth, which hinders early intervention with sFGR and fetal brain injury in clinical settings.

Metabolic profiles reflect the state of individual physiology, presence of diseases, or other conditions [8]. Pregnancy is a special physiological state accompanied by numerous maternal physiological adjustments, such as altered production of metabolic molecules, proteins, immune factors, and other dynamic adjustments for fetal growth and nervous system development [9–13]. Abnormal metabolic fluctuations in the maternal plasma usually indicate physiological anomality in the fetal environment in utero [14], leading to adverse outcomes such as preeclampsia (PE) and fetal growth restriction (FGR) [14, 15]. Many studies have focused on the metabolomic characteristics of fetal samples at birth [16, 17], which provides a limited reference value for early diagnosis and intervention in clinical practice. To date, eight longitudinal cohort studies of singleton pregnancies have demonstrated unique advantages in characterizing fetal development or disease dynamics during pregnancy or in predicting pregnancy outcomes [14, 18–24]. Intriguingly, previous research has shown that the maternal metabolic clock is closely correlated with the time of delivery [22] and FGR [14], which is more accurate than ultrasonography findings [22]. Additionally, metabolic abnormalities in the second trimester show potential for the identification of biomarkers related to preterm birth [25]. However, only a few cross-sectional studies have been performed to profile the metabolomic characteristics of intrapartum or postpartum sFGR samples [16, 17, 26, 27], and the metabolic dynamics of MCDA twins have not been reported. Therefore, the metabolic dynamics of twins during pregnancy, especially in the first and second trimester, are not well understood, which hinders the application of the metabolome for the early prediction of sFGR and fetal brain injury in the perinatal period, let alone the early understanding of the long-term physical and neurological development of MCDA fetuses.

**Fig. 1:**
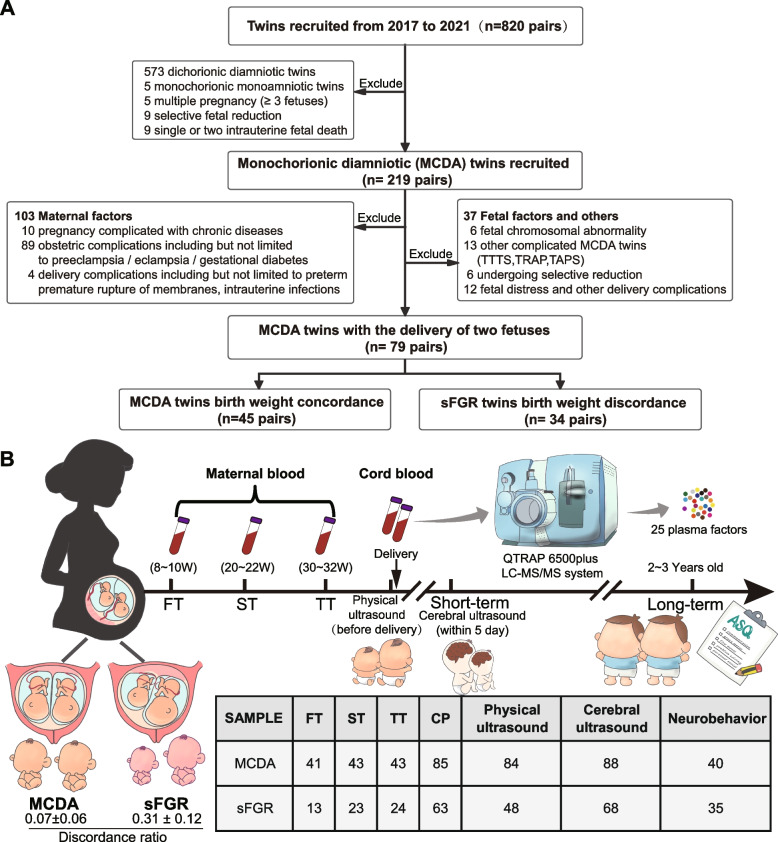
Flowchart of the study design, population, and experiment. **A** Flowchart of volunteer recruitment and grouping. **B** The cohort of 79 MCDA twin pregnancies was divided into two groups based on discordance of fetal birth weight. The number of samples in each twin group is listed in the table for metabolomics, ultrasound results and follow-up survey. TTTS, twin to twin transfusion syndrome; TRAP, twin reversed arterial perfusion; TAPS, twin anemia-polycythemia sequence; sFGR, selective fetal growth restriction; MCDA, normal monochorionic diamniotic twins; FT, first trimester, ST, second trimester, TT, third trimester; CP, cord plasma

To address these concerns, we quantified 25 key plasma metabolites in maternal plasma samples from all three trimesters and cord plasma in MCDA twins and dissected the relationship among these metabolites, sFGR, and adverse neurological outcomes from short-term and long-term perspectives (Fig. 1), with the aim of permitting possible early prediction and intervention for sFGR and fetal brain injury.

## Methods

### Study design and population

This was a prospective longitudinal cohort study of sFGR. Pregnant women with MCDA twin pregnancies who underwent cesarean section between September 2017 and December 2021 were recruited for this study. The inclusion criteria were MCDA twin pregnancies, cesarean delivery, and agreement to undergo follow-up after delivery. The exclusion criteria were maternal factors, including chronic diseases; obstetric and delivery complications; fetal factors, including major congenital anomalies; and other complicated MCDA twin pregnancies undergoing selective reduction and other delivery complications (Fig. 1A). In our study, chorionicity was determined using ultrasonography by two different obstetric sonographers at 6–14 weeks when twin pregnancies were independently detected. We followed the guideline recommendations which include the following criteria: (1) the number of gestational sacs at 6–9 weeks [28] and (2) the identification of the “T” sign of the amniotic-placental junction between the twins at 10–14 weeks [29–31]. After the twins were determined as MCDA twin, a board-certified twin-specialized obstetric sonographer was assigned to perform the ultrasonography examination and record fetal biometry/Doppler indices. sFGR was identified based on the diagnosis of the International Society of Ultrasound in Obstetrics and Gynecology (ISUOG) in 2016 that one fetus estimated fetal weight (EFW) < 10th centile and the intertwin weight discordance > 25% [32]. Finally, among 820 recruited twin pregnancies, 34 pairs of sFGR twins and 45 pairs of uncomplicated MCDA twin pregnancies were included in this study.

Maternal and fetal clinical characteristics, including maternal age, body mass index (BMI) before pregnancy and delivery, gestational week, fetal birth weight and height, sex, 1- and 5-min Apgar scores, and umbilical blood pH, were measured and recorded within 24 h after delivery (Table 1). Before delivery, fetal growth indicators, including the head circumference (HC), abdominal circumference (AC), biparietal diameter (BPD), and femur length (FL), were determined using ultrasound measurements (84 cases in the MCDA group and 48 in the sFGR group) (Additional file 1: Table S1). Additionally, within 5 days after delivery, data from neonatal cerebral ultrasonography (88 cases in the MCDA group, 68 in the sFGR group), including abnormal signal in brain, choroid plexus, and white matter, were collected to identify signs of neonatal brain injury (Additional file 1: Table S2). Two fixed obstetric sonographers conducted ultrasonography and determined the conditions of fetal growth and neonatal brain injury.

**Table 1:**
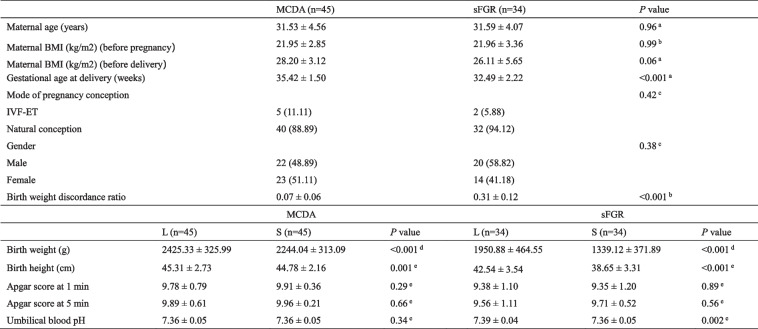
Clinical characteristics of MCDA and sFGR twins

Continuous variables were presented as the mean ± standard deviation (SD) and categorical variables were presented as numbers (percentages). *P* < 0.05 was considered statistically significant

*MCDA* normal monochorionic diamniotic twins, *sFGR* selective fetal growth restriction, *L* the larger fetus, *S* the smaller fetus, *BMI* body mass index, *IVF-ET* in vitro fertilization-embryo transfer

^a^Student’s *t*-test

^b^Mann–Whitney *U* test

^c^Chi-square test

^d^Paired *t*-test

^e^Wilcoxon’s-sign-rank-test

### Follow-up neurocognitive behavioral development surveys

The neurocognitive behavioral development of infants aged 2–3 years was assessed using the Ages and Stages Questionnaire-third edition subscale (ASQ-3), which included the first speaking and walking times and five developmental domains: communication, gross motor, fine motor, problem solving, and personal social, with a total of 60 points available in the combined evaluation for each infant [33]. According to the suggestion provided by the World Health Organization (WHO) for anthropometric analysis of children younger than 5 years of age, *z*-scores were calculated using the “anthro_zscores” function in R package “anthro” (version 1.0.0): (1) “Height for age” is used to evaluate whether a child’s height is within the normal range for their age group; (2) “Weight for age” is used to assess whether a child’s weight falls within the expected range for their age; (3) “Height for weight” is used to assess the current nutritional or developmental status of a child; (4) “BMI for age” is used to assess whether the proportion of a child’s weight to their height is within the appropriate range. All follow-up surveys were conducted via telephone or online questionnaires, with oral responses.

### Sample collection

Peripheral blood samples were collected and centrifuged at 1650 rcf (g) for 10 min at 4 °C, and plasma was collected and stored at – 80 °C. The peripheral blood of pregnant women in the first trimester was collected at 8–10 weeks (41 cases in the MCDA group, 13 in the sFGR group), that in second trimester was collected at 20–22 weeks (43 cases in the MCDA group, 23 in the sFGR group), and that in third trimester was collected at 30–32 weeks (43 cases in the MCDA group, 24 in the sFGR group). Cord blood was drawn quickly after the fetus was delivered (85 cases in the MCDA group, 63 in the sFGR group). All samples underwent only one freeze–thaw cycle prior to detection.

### QTRAP® 6500 plus LC–MS/MS targeted metabolomics

A total of 337 plasma samples, including maternal plasma (*n* = 187) and cord plasma (*n* = 150), were used to quantify the levels of the 25 target metabolites. The number of metabolites detected in each sample is shown in Figure S1 in Additional file 1. Plasma (20 μL) was aliquoted into a 1.5-mL Axygen tube and mixed with 80 μL of internal standards in methanol. The metabolites in the samples were precipitated by vortexing for 1 min at 4–8 °C, the supernatant was recovered by centrifugation at 20,000 g at 4 °C for 10 min, and 1 μL was injected into the system (QTRAP® 6500 plus LC–MS/MS system, SCIEX, Foster City, CA, USA). The reagents and resources used are listed in Table S3 in Additional file 1.

For amino acid (AA) measurements, including l-alanine, l-serine, l-proline, l-valine, l-threonine, l-lysine, l-glutamic acid, l-methionine, l-histidine, l-phenylalanine, l-arginine, l-tyrosine, l-cystine, l-leucine, and l-isoleucine, the supernatants (3 μL) were analyzed by injection onto an Intrada Amino Acid UPLC column (100 × 3 mm i.d., 3 μm; Imtakt, Kyoto, Japan) at a flow rate of 0.5 mL/min using the LC-20AD Shimadzu pump system and SIL-20AXR autosampler interfaced with the API 6500Q-TRAP mass spectrometer (SCIEX, Framingham, MA). The analytes were monitored using electrospray ionization in negative-ion mode with multiple reaction monitoring (MRM) of the precursor and characteristic production transitions of AAs.

For the phenylacetylglutamine (PAGln) pathway measurement, including PAGln and hippuric acid, supernatants (1 μL) were analyzed by injection onto a Kinetex C18 column (50 mm × 2.1 mm, 2.6 μm; Phenomenex, Torrance, CA) at a flow rate of 0.5 mL/min using the LC-20AD Shimadazu pump system and SIL-20AXR autosampler interfaced with the API 6500Q-TRAP mass spectrometer. The analytes were monitored using electrospray ionization in negative ion mode with MRM of the precursor and characteristic production transitions of the PAGln pathway.

For the trimethylamine-N-oxide (TMAO) pathway measurement, including carnitine, TMAO, betaine, choline, butyrobetaine, trimethyllysine, N, N, N-trimethyl-5-aminovaleric acid (TMVAV), and creatinine, the supernatants (3 μL) were analyzed by injection onto a silica column (2.0 × 150 mm; Luna 5u Silica 100A, Phenomenex, Torrance, CA) at a flow rate of 0.5 mL/min using the LC-20AD Shimadzu pump system and SIL-20AXR autosampler interfaced with the API 6500Q-TRAP mass spectrometer. The analytes were monitored using electrospray ionization in negative-ion mode with MRM of the precursor and characteristic production transitions of the TAMO pathway.

### Statistical analysis

Student’s *t*-test was used to determine the statistical significance in the intergroup comparison of normally distributed data (if *P* > 0.05 in the Shapiro–Wilk normality test), whereas the Mann–Whitney *U* test was used for nonparametric comparisons of data between two different groups (if *P* < 0.05 in the Shapiro–Wilk normality test). The paired *t*-test and Wilcoxon’s-sign-rank-test were used for comparisons between large and small fetuses among twins. After correcting for multiple testing, a 2-sided *P* value of less than 0.002 (0.05/25) was used to indicate evidence of association. The chi-square test was used to compare the proportion of categories between the sFGR and MCDA groups in Table 1. We conducted mixed-effects logistic regression models using the function the function ‘glmer’ in the R package “lme4” (version 1.1–27) [34] to evaluate the statistical correlation between sFGR and the parameters of fetal brain injury estimated by ultrasonography (Additional file 1: Table S2). These models all include a fixed component for sFGR status and a random component for twin pairs to comprehensively assess the effect of sFGR and the relatedness of the individuals within twin pairs. Spearman’s-based correlation test was used to determine the correlation coefficient and *P* value between any two objects, using the functions “cor.test” and “cor” in the R package “stats” (version 3.6.0). Partial least squares discriminant analysis (PLS-DA) was performed to evaluate the plasma metabolome profiles in maternal plasma samples during pregnancy and cord plasma samples via the function “plsda” in the R package “mixOmics” (version 6.20.0) [35]. Binomial logistic regression analysis was conducted based on the Log2-transformed absolute concentration(s) of individual or multiple selected intergroup significantly differential metabolites using the function “glm” in the R package “stats” (version 3.6.0). The optimal combination of metabolites with the lowest Akaike information criterion (AIC) value in fitting new regression models was further determined using the function “stepAIC” in the R package “MASS” (version 7.3–58.1) [36], with a backward selection strategy. The predictions of a specific model were obtained using the function “Predict” in the R package “car” (version 3.0–10) [37]. Then, the function “roc” and “ggroc” in the R package “pROC” (version 1.17.0.1) [38] were used to build the receiver operating characteristic (ROC) object to evaluate the capacity of metabolites to identify sFGR or fetal brain injury. The 95% confidence interval (CI) of area under the curve (AUC) was calculated using the function “ci.auc” with the default “delong” method in the pROC package [38]. The ROC curves between Model A and B were compared using the function "roc.test" in the pROC package (version 1.17.0.1) with the bootstrap method and the number of bootstrap replicates was set as 10,000 [38]. The three ROC curves built based on the significantly differential metabolites identified in each trimester were compared among each other using function "roc.test" in the pROC package (version 1.17.0.1) with the default parameter “delong” method [38]. The linear mixed-effects model (LMM) were applied to assess the impact of trimesters and disease status (sFGR vs. MCDA or fetal brain injury (abnormal) vs. normal), and their interaction on peripheral metabolite levels employing the ‘lmer’ function in the R package ‘lme4’ (version 1.1–27) [34]. A *P* value < 0.05 was considered statistically significant (ns: *P* ≥ 0.05; ∗ *P* < 0.05; ∗  ∗ *P* < 0.01; ∗  ∗  ∗ *P* < 0.001).

## Results

### Clinical characteristics of pregnant women with or without sFGR

A total of 45 cases from the MCDA group and 34 cases from the sFGR group were included in our study cohort. Maternal plasma samples were collected during different trimesters, whereas cord plasma samples were collected during delivery (Fig. 1A, Table 1). Based on the important role in sFGR emphasized in our previous research and literature reports on untargeted metabolomics, 25 key metabolites were absolutely quantified by targeted metabolomics that were closely related to fetal growth and development [26, 39], vascular system [17, 40–42], and nervous system damage [16, 27, 43, 44]. To explore the association between maternal and fetal metabolite levels and the long-term neurocognitive behavioral development of infants, we also followed up with infants at the age of 2–3 years using the ASQ3 (Fig. 1B).

There were no significant differences between the MCDA and sFGR groups in maternal age (MCDA: 31.53 ± 4.56 years; sFGR: 31.59 ± 4.07 years), maternal BMI before pregnancy (MCDA: 21.95 ± 2.85 kg/m^2^; sFGR: 21.96 ± 3.36 kg/m^2^), maternal BMI before delivery (MCDA: 28.20 ± 3.12 kg/m^2^; sFGR: 26.11 ± 5.65 kg/m^2^), in vitro fertilization and embryo transfer pregnancy rate (MCDA: 11.1%; sFGR: 5.9%), and fetal sex (MCDA: 48.9%; sFGR: 58.8%). Moreover, the gestational weeks decreased significantly (MCDA: 35.42 ± 1.50 weeks; sFGR: 32.49 ± 2.22 weeks, *P* < 0.001), whereas the weight inconsistency rate was increased notably in sFGR (MCDA: 0.07 ± 0.06; sFGR: 0.31 ± 0.12,* P* < 0.001). Fetal birth weight (MCDA-small [S]: 2244.04 ± 313.09 g; sFGR-S: 1339.12 ± 371.89 g) and height (MCDA-S: 44.78 ± 2.16 cm; sFGR-S: 38.65 ± 3.31 cm) were significantly decreased in the smaller fetuses compared with the larger fetuses (birth weight: MCDA-large [L]: 2425.33 ± 325.99 g, *P* < 0.001; sFGR-L: 1950.88 ± 464.55 g,* P* < 0.001; birth height: MCDA-L: 45.31 ± 2.73 cm,* P* = 0.001; sFGR-L: 42.54 ± 3.54 cm,* P* < 0.001). Additionally, there was no significant difference in 1- and 5-min Apgar scores in either the MCDA or sFGR group between the larger and the smaller fetuses. However, the cord blood pH in sFGR was significantly different between the larger and the smaller fetuses (sFGR-L: 7.39 ± 0.04; sFGR-S: 7.36 ± 0.05,* P* = 0.002) (Table 1).

### Anomalous maternal–fetal metabolic level of sFGR

**Fig. 2:**
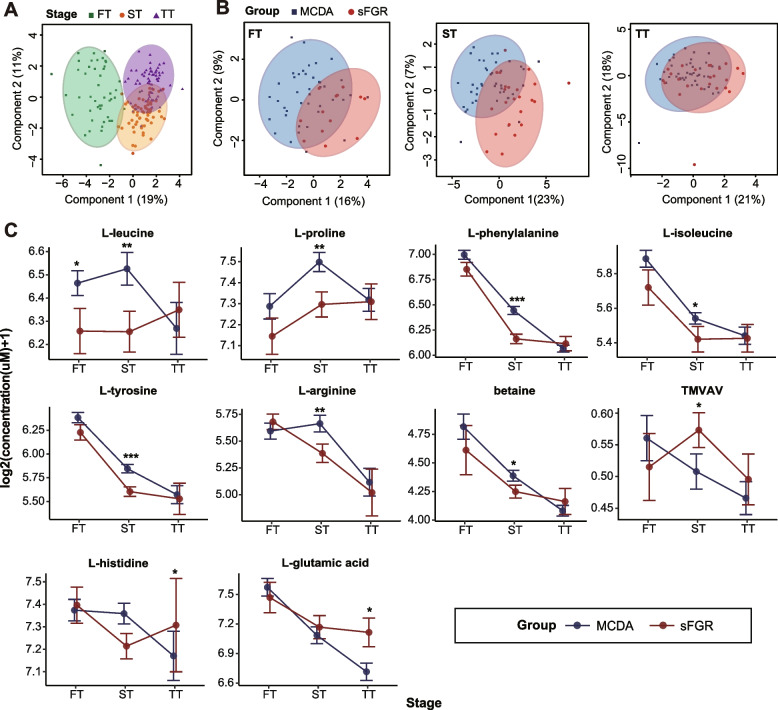
Maternal metabolite levels in different trimesters between sFGR and MCDA. **A** PLS-DA of the maternal plasma metabolome in the different trimesters. **B** PLS-DA of the maternal plasma metabolome between the sFGR and MCDA groups in the first, second, and third trimester. Component 1: the set of coefficients that produces component scores that have the biggest difference between experimental factors. Component 2: the second set of coefficients that explains the second most variability between experimental factors. Circles in PLS-DA represent 95% confidence intervals (CI); each point in the figure represents a sample, and samples in the same group are represented by the same color. **C** Line plot showing the maternal plasma differential metabolite levels in different trimesters between sFGR and MCDA. Student’s *t*-test and Mann–Whitney *U* test were used for parametric or nonparametric comparisons in two groups. PLS-DA, partial least squares discriminant analysis; MP, maternal plasma; FT, first trimester, ST, second trimester, TT, third trimester; sFGR, selective fetal growth restriction; MCDA, normal monochorionic diamniotic twins; *TMVAV*, N, N, N-trimethyl-5-aminovaleric acid; Log2-transformed metabolite concentrations (μM) are presented as the mean ± standard deviation (SD). *P < 0.05, **P < 0.01, ***P < 0.001

PLS-DA based on the level of 25 metabolites showed that maternal plasma samples in the three trimesters were apparently separated well (Fig. 2A, Table 2, Additional file 1: Figure S2A and Table S4), which is consistent with findings of previous reports that maternal metabolite levels vary in different trimesters [22]. With an increase in gestational weeks, we observed a consistent trend between the MCDA and sFGR groups: nine metabolites were gradually decreased, two metabolites were gradually increased, and nine metabolites showed a notable increasing or decreasing trend in the second trimester (Fig. 2C, Table 2, Additional file 1: Figure S3 and Table S4). Additionally, five metabolites, including l-threonine, l-histidine, l-cystine, l-leucine, and TMVAV, showed a contrasting trend between the MCDA and sFGR groups (Fig. 2C, Table 2, Additional file 1: Figure S3 and Table S4). Specifically, the maternal plasma metabolites in the second trimester showed the most distinct separation between the sFGR and MCDA groups (Fig. 2B). l-leucine was the only significantly different metabolite whose level decreased in the sFGR group in the first trimester, while the levels of l-glutamic acid and l-histidine in the third trimester were notably increased in the sFGR group compared to the MCDA group (Fig. 2C). Up to eight metabolites showed significant intergroup differences between the MCDA and sFGR groups in the second trimester. The levels of l-proline, l-phenylalanine, l-arginine, l-tyrosine, l-leucine, l-isoleucine, and betaine were significantly decreased, while the level of TMVAV was significantly increased in the sFGR group (Fig. 2C, Table 2). Among these, the *P* values of l-phenylalanine and l-tyrosine were less than 0.002 (0.05/25) (Table 2).

**Table 2:**
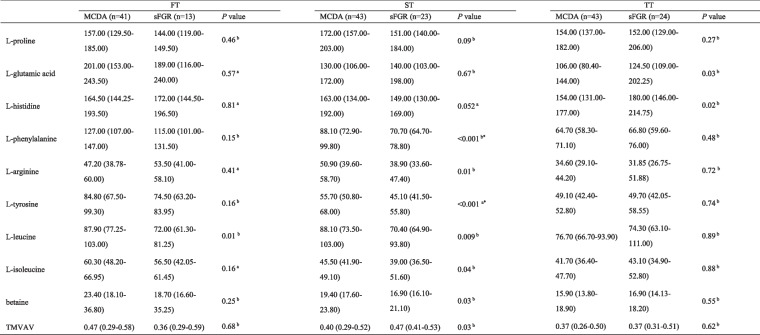
Differential metabolite concentrations in maternal plasma during pregnancy quantified by targeted metabolomics

* FT* first trimester, *ST* second trimester, *TT* third trimester, *MCDA* normal monochorionic diamniotic twins, *sFGR* selective fetal growth restriction, *TMVAV*, N, N, N-trimethyl-5-aminovaleric acid

^a^Student’s *t*-test

^b^Mann–Whitney *U* test

Data are presented as median and interquartile range (25–75%) of measured concentrations (μM). *P* < 0.05 was considered statistically significant

^*^Presented as the corrected *P* < 0.002

**Fig. 3:**
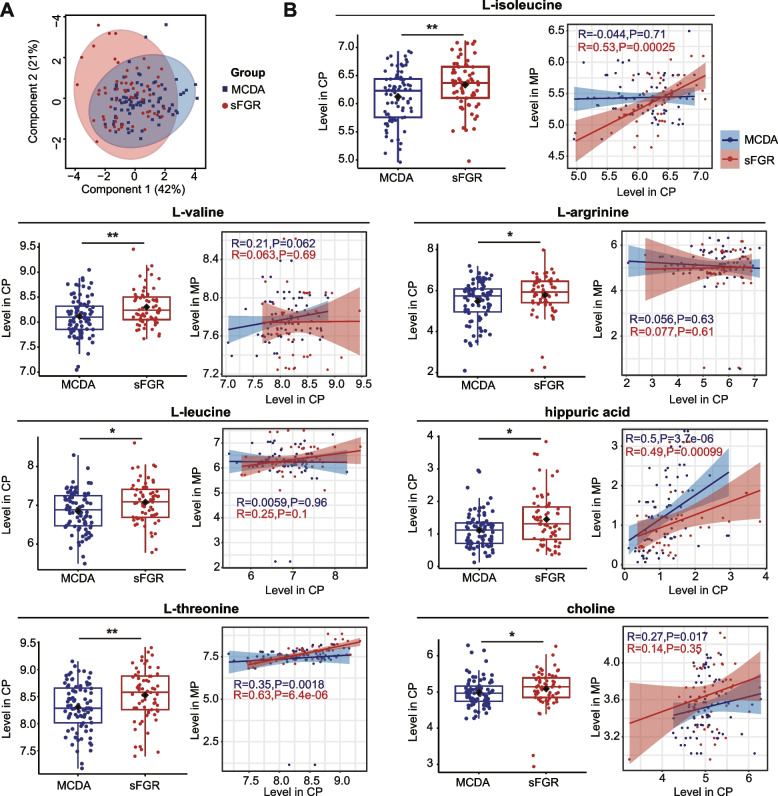
Fetal metabolite levels between sFGR and MCDA. **A** PLS-DA of the cord plasma metabolome between the sFGR and MCDA groups. Component 1: the set of coefficients that produces component scores that have the biggest difference between experimental factors. Component 2: the second set of coefficients that explains the second most variability between experimental factors. Circles in PLS-DA represent 95% confidence intervals (CI); each point in the figure represents a sample, and samples in the same group are represented by the same color. PLS-DA, partial least squares discriminant analysis. **B** Box plot showing the cord plasma metabolite levels between the sFGR and MCDA groups and their correlation with the maternal plasma metabolite levels in the third trimester. Mann–Whitney *U* tests were used for nonparametric comparisons between two groups. The metabolite levels in cord plasma and maternal plasma were determined as Log2-transformed metabolite concentrations (μM). Data were presented as the median and interquartile range (25–75%). The correlation between the metabolite levels in cord plasma and maternal plasma in the third trimester was examined using Spearman’s correlation, and Spearman’s correlation coefficients (*R*) were used to present the correlation between the two groups. The shadow around the linear regression trendline shows the 95% CI. sFGR, selective fetal growth restriction; MCDA, normal monochorionic diamniotic twins; CP, cord plasma; MP, maternal plasma; *P < 0.05, **P < 0.01

PLS-DA showed an unsatisfactory separation trend in cord plasma (Fig. 3A). However, up to seven differential metabolites, including l-valine, l-threonine, l-isoleucine, l-leucine, l-arginine, choline, and hippuric acid, were notably elevated in the sFGR group (Fig. 3B, Additional file 1: Table S5). Among the seven differential metabolites identified, l-threonine, l-isoleucine, choline, and hippuric acid also presented significantly positive maternal–fetal correlations between cord plasma and maternal plasma in the third trimester (Fig. 3B). Additionally, based on these 25 metabolites, significantly different metabolite was not observed in cord plasma between large and small fetuses in the sFGR group (Additional file 1: Table S5).

### Relationship between maternal–fetal plasma metabolites and fetal growth

**Fig. 4:**
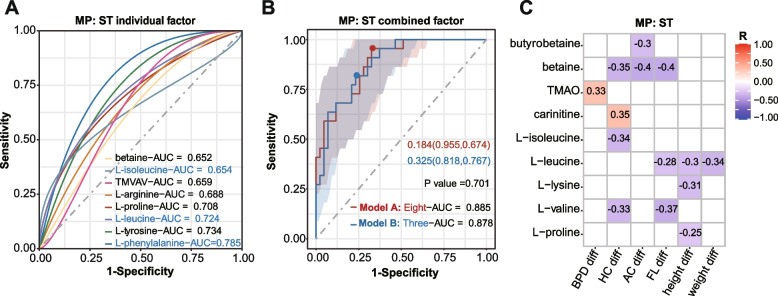
Maternal metabolites in the second trimester to predict sFGR. **A** ROC curves of models constructed based on each significantly differential metabolite in the second trimester for the prediction of sFGR. **B** ROC curves of models constructed based on the combination of significantly differential metabolites for the prediction of sFGR. Eight-AUC presents the predictive effectiveness of the combination of eight significant differential metabolites, including l-proline, l-arginine, l-tyrosine, l-leucine, l-isoleucine, l-phenylalanine, betaine, and TMVAV. Three-AUC presents the predictive effectiveness of optimized combination of three significant differential metabolites, including l-leucine, l-isoleucine, and l-phenylalanine. The dots on the broken lines in **B** represent the best cut-off value, and the values in the figure represent the best cut-off value (sensitivity, specificity). The shadow around the linear regression trend line shows the 95% confidence interval (CI); The *P* value in **B** was shown as the significant difference between model A and model B; *P* < 0.05 was considered statistically significant. **C** The correlation between metabolite levels in the second trimester and the difference in physical development parameters of twins were examined by Spearman correlation analysis. Spearman’s correlation coefficients (*R*) were used to present the correlations, and only *R* values with *P* < 0.05 were plotted. sFGR, selective fetal growth restriction; ST, second trimester; TMVAV, N, N, N-trimethyl-5-aminovaleric acid; TMAO, trimethylamine-N-oxide; HC, head circumference; AC, abdominal circumference; BPD, biparietal diameter; FL, femur length; S/D, ratio of fetal umbilical artery systolic pressure to diastolic pressure; ROC, receiver operating characteristic; AUC, area under curve; diff, the difference between the larger and the smaller fetus

In the clinical setting, ultrasonography is used to evaluate intrauterine fetal development before delivery. Fetal development indicators including HC (MCDA: 29.69 ± 4.12 cm; sFGR: 28.34 ± 2.73 cm,* P* < 0.001), AC (MCDA: 28.34 ± 4.29 cm; sFGR: 25.83 ± 4.03 cm,* P* < 0.001), BPD (MCDA: 8.21 ± 1.21 cm; sFGR: 7.87 ± 0.87 cm,* P* < 0.001), FL (MCDA: 6.24 ± 1.01 cm; sFGR: 5.71 ± 0.82 cm,* P* < 0.001), and FL/BPD (MCDA: 0.76 ± 0.05; sFGR: 0.72 ± 0.05,* P* < 0.001) were significantly lower in the sFGR group than in the MCDA group (Additional file 1: Table S1). To further evaluate the efficacy of differential metabolites between sFGR and MCDA in predicting sFGR globally, logistic regressions were constructed based on individual metabolites or a combination of the differential metabolites in first trimester, second trimester, and third trimester, separately (Fig. 4A–B, Additional file 1: Figure S4A–C). Specifically, based on the aforementioned differential metabolites (*P* < 0.05), the predictive efficiencies in first trimester (area under the curve [AUC]: model A [l-leucine], 0.793) and third trimester (AUC: model A [l-glutamic acid and l-histidine], 0.744) were less than 0.8, and the highest predictive efficiency was obtained in second trimester (AUC: model A [l-proline, l-phenylalanine, l-arginine, l-tyrosine, l-leucine, l-isoleucine, betaine, and TMVAV], 0.885; model B [l-phenylalanine, l-leucine, and l-isoleucine], 0.878) (Fig. 4A–B, Additional file 1: Figure S4A–C and Table S6). In addition, we compared the ROC curves based on maternal plasma metabolite levels in different trimesters, and the result showed that the performance of the model based on the second trimester was significantly better than that of the third trimester (*P* = 0.036) (Additional file 1: Figure S4D). In cord plasma, ROC curves were constructed by differential metabolites between the sFGR and MCDA groups. Remarkably, the predictive efficiency (AUC: model A [l-valine, l-threonine, l-isoleucine, l-leucine, choline, and hippuric acid], 0.715; model B [l-valine, l-isoleucine, and hippuric acid], 0.702) in cord plasma (Additional file 1: Figure S4E–F and Table S6) was lower than that in all trimesters in maternal plasma. Additionally, there was no significant difference between models A and B neither for maternal plasma metabolites in second trimester nor for cord plasma (Fig. 4B, Additional file 1: Figure S4F).

Clinically, MCDA twins with a higher rate of discordance often have more serious adverse outcomes, including intrauterine fetal death [7, 45]. Hence, we performed a correlation analysis between maternal metabolites and the differences (diff) in physical development parameters of pairs of twins. Our findings revealed that l-leucine in the first trimester was negatively correlated with height diff (*R* =  − 0.32, *P* = 0.02) (Additional file 1: Figure S5B, Additional file 2: Table S7). l-glutamic acid in the third trimester was positively correlated with weight diff (*R* = 0.25, *P* = 0.05) (Additional file 1: Figure S6C, Additional file 2: Table S7). Moreover, in the second trimester, l-leucine was significantly negatively correlated with FL diff (*R* =  − 0.28, *P* = 0.04), height diff (*R* =  − 0.30, *P* = 0.02), and weight diff (*R* =  − 0.34, *P* = 0.007), and l-isoleucine was negatively correlated with HC diff (*R* =  − 0.34, *P* = 0.01) (Fig. 4C, Additional file 2: Table S7). l-phenylalanine in the second trimester was significantly positively correlated with birth weight (L: *R* = 0.28, *P* = 0.02; S: *R* = 0.32, *P* = 0.009), BPD (L: *R* = 0.34, *P* = 0.01; S: *R* = 0.29, *P* = 0.04), HC (L: *R* = 0.36, *P* = 0.009; S: *R* = 0.38, *P* = 0.005), and FL (L: *R* = 0.28, *P* = 0.04; S: *R* = 0.32, *P* = 0.02) in the large and small fetuses in the sFGR group (Additional file 1: Figure S6A, Additional file 2: Table S7). In addition, we found that seven elevated differential metabolites in cord plasma in the sFGR group were significantly negatively correlated with different fetal physical development parameters (Fig. 3B, Additional file 1: Figure S7, Additional file 2: Table S7).

**Fig. 5:**
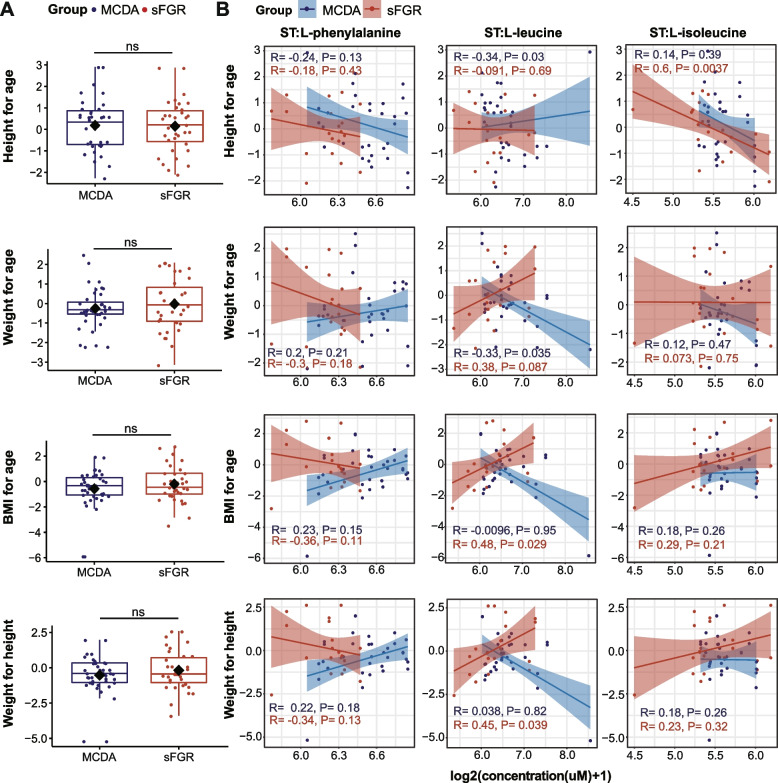
Correlation between metabolites in the second trimester and physical development later in life. **A** Box plot showing the comparison of height for age, weight for age, BMI for age, and height for weight between the sFGR and MCDA groups. The Mann–Whitney *U* test was used for nonparametric comparisons between the sFGR and MCDA groups. **B** The correlation between the metabolites in the second trimester and the standardized development indicators were examined by Spearman correlation analysis. Spearman’s correlation coefficients (*R*) were used to present the correlations. The shadow around the linear regression trendline shows the 95% confidence interval (CI). The metabolite level in **B** was determined as Log2-transformed metabolite concentrations (μM); sFGR, selective fetal growth restriction; MCDA, normal monochorionic diamniotic twins; ST, second trimester; CP, cord plasma; MP, maternal plasma;* P* < 0.05 was considered statistically significant. ns, nonsignificant

To investigate potential associations between early-life metabolic alterations and later physical development, we analyzed the correlation between the level of maternal–fetal metabolites and evaluation indices of physical development in infants aged 2–3 years after birth. There were no significant differences in the age-standardized weight, height, BMI, and weight for height between the sFGR and MCDA groups (Fig. 5A). However, l-leucine in the second trimester was significantly related to BMI for age (*R* = 0.48, *P* = 0.029) and weight for height (*R* = 0.45, *P* = 0.039), whereas l-isoleucine in the second trimester was significantly positively correlated with height for age (*R* = 0.6, *P* = 0.0037) in the sFGR group (Fig. 5B).

### Relationship between maternal–fetal plasma metabolites and neurocognitive behavioral development

**Fig. 6:**
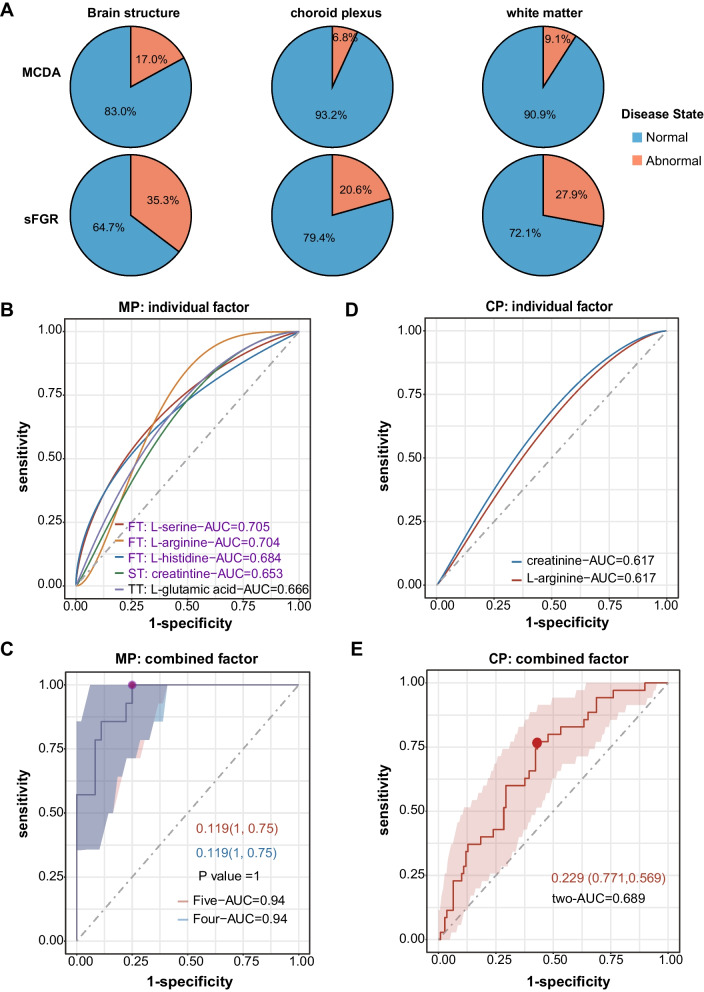
Maternal–fetal metabolites in the evaluation of fetal brain injury in MCDA twins. **A** Pie plot showing the proportion of abnormal signal in the brain structure, white matter, and choroid plexus, detected by cerebral ultrasound, in either the sFGR (*n* = 68) or MCDA (*n* = 88) group. **B** ROC curves of models constructed based on each significantly differential maternal metabolites for the prediction of fetal brain injury. **C** ROC curve of models constructed based on the combination of significantly differential maternal metabolites for the prediction of fetal brain injury. The five-AUC presents the predictive effectiveness of the combination of five metabolites, including l-serine, l-histidine, and l-arginine in the first trimester, creatinine in the second trimester, and l-glutamic acid in the third trimester. The four-AUC presents the predictive effectiveness of the optimized combination of four metabolites, including l-serine, l-histidine, and l-arginine in the first trimester, creatinine in the second trimester. **D** ROC curves of models constructed based on each significantly differential cord plasma metabolite for the prediction of fetal brain injury. **E** ROC curve of models constructed based on the combination of significantly differential cord plasma metabolite for the prediction of fetal brain injury. The two-AUC presents the predictive effectiveness of the combination of two metabolites, including l-arginine and creatinine in cord plasma. The dots on the broken lines in **C** and **E** represent the best cut-off value, and the values in the figure represent the best cut-off value (sensitivity, specificity). The shadow around the linear regression trendline shows the 95% confidence interval (CI). The *P* value in **C** and **E** were shown as the significant difference between model A and model B; sFGR, selective fetal growth restriction; MCDA, normal monochorionic diamniotic twins; FT, first trimester, ST, second trimester, TT, third trimester; ROC, receiver operating characteristic; AUC, area under curve; CP, cord plasma; MP, maternal plasma; *P* < 0.05 was considered statistically significant

Neonatal brain injury is a serious adverse outcome in MCDA twins, and those with sFGR suffer from higher morbidity and severity [1, 7, 46, 47]. Consistent with this, the incidence of brain injury in the sFGR group was higher than that in the MCDA group according to the cerebral ultrasonography results of all neonates in the cohort, including abnormal brain structure, choroid plexus, and white matter damage (Fig. 6A). The mixed-effects logistic regression analysis further revealed that the occurrence of sFGR were significantly associated with the abnormity of choroid plexus (*P* = 0.040), while not with abnormal brain structure (*P* = 0.062) and white matter damage (*P* = 0.141) (Additional file 1: Table S2). To explore the metabolites associated with the occurrence of fetal brain injury, samples were grouped into fetal brain injury or not. Intergroup comparison analysis revealed that l-serine and l-histidine in the first trimester, creatinine in the second trimester, and cord plasma were significantly decreased, while l-arginine in the first trimester, l-glutamic acid in the third trimester, and l-arginine in cord plasma were significantly increased in the fetal brain injury group (Additional file 1: Figure S8A). Furthermore, logistic regressions to distinguish brain injury were constructed based on the differential metabolites identified in maternal plasma and cord plasma, respectively (Fig. 6B–E). The model for maternal plasma metabolites showed outstanding value in distinguishing fetal brain injury (AUC: model A [l-serine, l-arginine, and l-histidine in first trimester, creatinine in second trimester, and l-glutamic acid in third trimester], 0.94; model B [l-serine, l-arginine, and l-histidine in first trimester, and creatinine in second trimester], 0.94) (Fig. 6B–C, Additional file 1: Table S8). Although l-arginine and creatinine were used in the maternal plasma and cord plasma models to predict fetal brain injury, the model in cord plasma (AUC: model A [l-arginine and creatinine in cord plasma], 0.689) showed low accuracy (Fig. 6B–E, Additional file 1: Table S8). Additionally, increased concentrations of l-glutamic acid in the third trimester were observed in the sFGR and fetal brain injury groups (Fig. 2C, Additional file 1: Figure S8A). We also found that the maternal plasma metabolites in the second trimester between the sFGR and MCDA groups showed suboptimal values in distinguishing fetal brain injury (AUC: model A, 0.762; model B [l-phenylalanine and l-arginine in second trimester], 0.732) (Additional file 1: Figure S8B–C and Table S8).

**Fig. 7:**
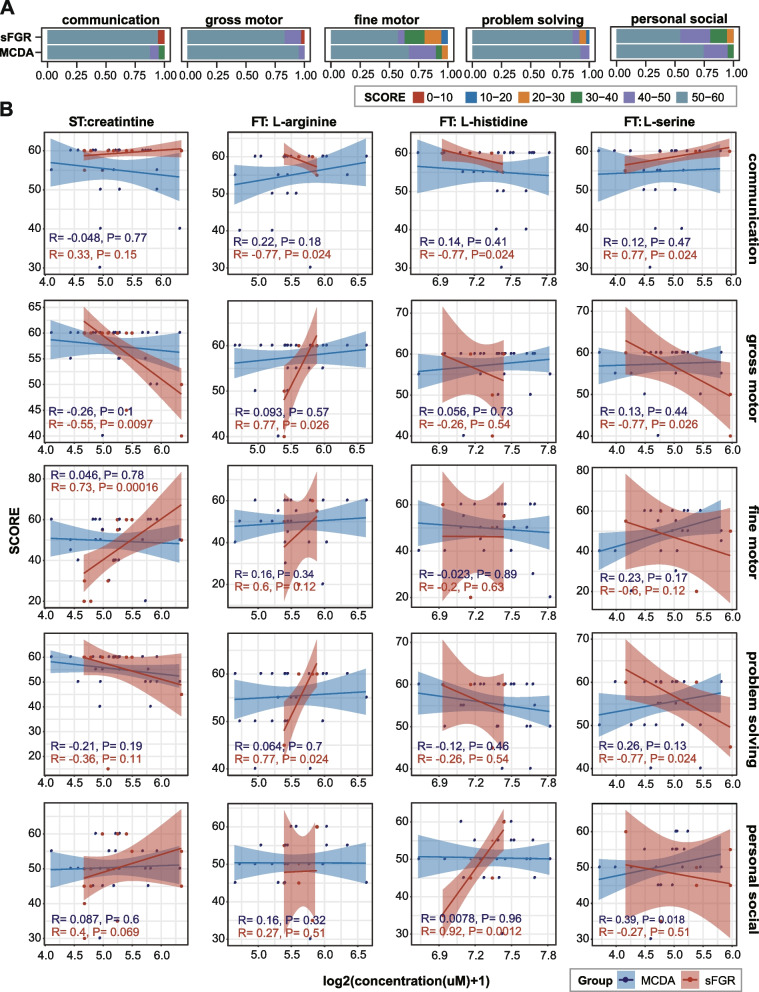
Correlation between maternal metabolites and neurocognitive behavioral development later in life in offspring. **A** Bar plot showing the percentage of different classes of scores in sFGR and MCDA groups for each developmental domain of the Ages and Stages Questionnaires third edition subscale (ASQ-3). **B** The correlation between the four metabolite concentrations and five evaluation indices of neurocognitive behavioral development were examined by Spearman correlation analysis. Spearman’s correlation coefficients (*R*) were used to present the correlation. The shadow around the linear regression trendline shows the 95% confidence interval (CI). The metabolite level in **B** was determined as Log2-transformed metabolite concentrations (μM); sFGR, selective fetal growth restriction; MCDA, normal monochorionic diamniotic twins; *P* < 0.05 was considered statistically significant

Furthermore, we compared the time of first talking and walking between the sFGR and MCDA groups for a simple assessment of neural and behavioral development, and found that the difference between the two groups was not statistically significant (Additional file 1: Figure S9A). However, the first walking time of infants with sFGR showed a significantly strong correlation with the levels of l-serine (*R* = 0.8, *P* = 0.018) and l-arginine (*R* =  − 0.8, *P* = 0.018) in the first trimester, as well as creatinine (*R* = 0.53, *P* = 0.013) in the second trimester (Additional file 1: Figure S9B). We evaluated neurocognitive behavioral development in infants by comparing the occurrence of low scores in each domain contained in the ASQ-3 between the sFGR and MCDA groups. The sFGR group showed a higher frequency of lower scores (< 30) in the five developmental domains than the MCDA group (Fig. 7A). For four differential maternal metabolites distinguishing fetal brain injury, the correlations between creatinine in the second trimester and gross motor (*R* =  − 0.55, *P* = 0.0097), fine motor (*R* = 0.73, *P* = 0.00016), l-arginine in the first trimester with communication (*R* =  − 0.77, *P* = 0.024), gross motor function (*R* = 0.77, *P* = 0.026), problem solving (*R* = 0.77, *P* = 0.024), l-histidine in first trimester with communication (*R* =  − 0.77, *P* = 0.024), personal social (*R* = 0.92, *P* = 0.001), l-serine in first trimester with communication (*R* = 0.77, *P* = 0.024), gross motor (*R* =  − 0.77, *P* = 0.026), and problem solving (*R* =  − 0.77, *P* = 0.024) in infants were high and significant (Fig. 7B). These findings provide important clues for the early diagnosis of fetal brain injury and long-term neurocognitive behavioral dysplasia in MCDA twins, especially in sFGR.

### Sensitivity analysis of maternal metabolites

Since the sample sizes of each trimester in our cohort were not completely consistent (Additional file 1: Figure S10A), to evaluate the potential impact of sample size on the results, we removed those cases with missing values for any metabolite, and retained 40 cases for sensitivity analysis (Additional file 1: Figure S10B; fetal growth restriction group: sFGR: *n* = 10, MCDA: *n* = 30; fetal brain injury group: abnormal: *n* = 12; normal: *n* = 28). Intergroup differential analysis between the sFGR and MCDA groups revealed that four original significant differential metabolites (l-proline, l-phenylalanine, betaine and TMVAV) were retained, and l-leucine was also close to the detection threshold (*P* = 0.055) (Additional file 1: Figure S10C, Additional file 3: Table S9). However, no significantly differential metabolites were detected in the third trimester. In the first trimester, we identified one original significantly differential metabolite: l-leucine, as well as three new significantly differential metabolites (l-proline, betaine and l-methionine) compared with the original cohort. Between the fetal brain injury and normal groups, two original differential metabolites (l-serine and l-arginine) in the first trimester were retained. l-valine was an additional differential metabolite added in the third trimester (Additional file 1: Figure S10C, Additional file 3: Table S9).

In addition, we also conducted a comparative evaluation on the prediction efficiency of the prediction model constructed by the original differential metabolites in different trimesters and the remaining 40 cases. Similar to the previous results, there were no significant differences between the first and third trimesters, whereas there were significant differences between the second and third trimesters (*P* = 0.016) (Additional file 1: Figure S10D). The ROC curve was also drawn on the prediction model constructed by the original four differential factors (l-serine, l-arginine, and l-histidine in the first trimester, and creatinine in second trimester) on the remaining 40 cases, and its AUC was as high as 0.899 (Additional file 1: Figure S10E). Linear mixed-effects models (LMMs) were used to explore the impact of individual heterogeneity and gestational stages on our results (Additional file 3: Table S9). A total of 21 metabolites were significantly different among three trimesters (*P* < 0.05), with only l-histidine, l-cystine, l-leucine, and TMVAV, showing no significant difference in expression levels with trimesters. Similar to previous observations in the original cohort, LMMs analysis revealed that l-histidine, l-leucine, and TMVAV showed no significant difference with trimesters (*P* > 0.05), while l-cystine had significant difference with trimesters in the fetal brain injury group (Additional file 3: Table S9).

Taken together, these findings provide important clues for the early diagnosis of fetal brain injury and long-term neurocognitive behavioral dysplasia in MCDA twins, especially in sFGR (Additional file 1: Figure S11).

## Discussion

According to the developmental origins of health and disease theory [48, 49], negative intrauterine experiences, such as growth restriction, may cause not only short-term but also permanent alterations in physiology and metabolism that increase the likelihood of adult diseases and health issues, particularly in the metabolic and nervous systems. In this study, we constructed the first longitudinal prospective sFGR cohort to date, covering long-term ASQ-3 follow-up results in infants aged 2–3 years, and quantified 25 key metabolites in 337 plasma samples from maternal plasma in three trimesters and cord plasma. We revealed dramatic changes in metabolites along different trimesters with the highest number of differential metabolites between sFGR and MCDA in second trimester. Moreover, specific combinations of differential metabolites were used to construct potential models to predict the occurrence of sFGR and fetal brain injury, which were also correlated with fetal growth indicators and long-term neurocognitive behavioral development. These findings highlight the fact that the predictive capability of maternal plasma differential metabolites in the first and second trimester is generally superior to that in the third trimester and cord plasma, and these metabolites may serve as useful biomarkers to develop strategies for the evaluation, prediction, diagnosis, and establishment of promising targets for early intervention with sFGR in clinical settings.

Recently, Liang et al. identified metabolites and metabolic pathways associated with pregnancy, provided a comprehensive view of metabolite changes during pregnancy and the postpartum period, and constructed a “metabolic clock” including five metabolites that accurately predicted gestational age and the timing of delivery [22]. As we have shown, maternal plasma metabolites during pregnancy have different expression patterns across the three gestational stages, suggesting that longitudinal cohort studies during pregnancy play a pivotal role in exploring the early prediction of fetal development and/or pregnancy disease. Our study and previous studies of singleton FGR suggested a clear association between metabolic profile alterations in maternal and cord blood [50]. However, only numerous cross-sectional metabolic analyses were performed in sFGR in previous studies, which mainly focused on the relationship between disease characteristics and variations in metabolism in intrapartum and postpartum samples, including maternal plasma [26], cord plasma [17, 26], placenta [17], meconium [27], and neonatal hair [16]. Owing to the lag in sample collection, the potential risks of sFGR could not be identified earlier, and as a result, its clinical application was limited in accessibility. Notably, we found that compared to cord plasma, the levels of metabolites in maternal plasma were better predictors of the occurrence of sFGR in our study, especially l-phenylalanine, l-leucine, and l-isoleucine in the second trimester. Identifying these metabolites in the second trimester advances the prediction window with strong predictive efficiency, which can improve the specificity of sFGR diagnosis in combination with ultrasonography during pregnancy.

Similar to a previous longitudinal cohort study, the second trimester has a unique metabolic tone during pregnancy [51], and abnormalities in fetal growth are often observed during this period. Studies have shown that the majority of l-phenylalanine is converted to l-tyrosine [52]. The lower levels of l-phenylalanine and l-tyrosine may also significantly affect placental angiogenesis [53], which may cause or aggravate placental dysfunction in twins with sFGR. Additionally, research has shown that the fetus receives considerable amounts of phenylalanine from the placenta, and two thirds of which are used for net protein synthesis [54]. This result suggested that the decrease in l-phenylalanine in maternal plasma during the second trimester may significantly affect the fetal protein synthesis process and thus participate in fetal growth restriction. l-leucine and l-isoleucine have been reported to be essential branched-chain amino acids (BCAAs) in the regulation of maternal antioxidative capacity and immune status, participate in early embryonic development through the activation of the mammalian target of rapamycin (mTOR) pathway to stimulate protein synthesis and cell growth [55–57], and can partially modulate the ability of proteins to translate messenger RNA (mRNA) [58]. Additionally, the decrease in BCAA concentrations may disrupt placental transportation [59], which may result in a reduced use rate of BCAAs in growth restricted fetuses [60]. Our results indicated a negative association between the reduction of leucine in the first trimester and second trimester, which are important windows of fetal development, and fetal growth. In addition to the metabolites included in the predictive model, we simultaneously identified several other metabolites such as l-arginine, TMAVA, methionine, and choline that may play a role in the occurrence of sFGR by regulating placental angiogenesis [61–63], oxidative stress [40, 64], and fetal growth [65].

Clinically, the incidence of brain injury in the FGR and sFGR groups was significantly higher than that in the control group [66], but the specific mechanism is still unclear. To date, there have been no reports on the use of metabolites to predict the occurrence of brain injury in one of the twins. To address these concerns, a predictive model including a combination of l-serine, l-histidine, and l-arginine in the first trimester, and creatinine in the second trimester was constructed for fetal brain injury assessment in one of the MCDA twins in our study. Pregnancy is a critical period for brain development in human embryos, and its developmental stages are divided into three periods [67, 68]. As our study shows, 75% (3/4) of the differential metabolites included in the predictive model were present in the first trimester. Generally, the neural tube is formed in the ectoderm in the first trimester and the neuroepithelial cells that form the neural tube produce neural progenitor cells and neurons [69]. Specifically, we found that the levels of l-serine and l-histidine were reduced in the brain injury group at an early stage. l-serine is the primary methyl donor to the one-carbon pool, and the methylation level affects brain development and function, such as neurotransmitter uptake, dopamine uptake involved in synaptic transmission, and dendritic spine architecture, which are linked to newborn attention and movement quality [70]. l-arginine is an indispensable precursor of nitric oxide, which plays an essential role in angiogenesis and ATP homeostasis that promote neural cells and blood vessels proliferation and differentiation when it extends neural and vascular processes [71]. Additionally, l-arginine can induce neuroinflammation via the two key enzymes: nitric oxide synthase and arginase, which may lead to nerve system damage [71, 72]. Histidine, as the precursor of carnosine, which acts as an antioxidant and scavenger of reactive oxygen species (ROS) [44, 73, 74], may also contribute to promoting oxidative stress in the placenta–brain axis of infants with sFGR. Meanwhile, histidine is crucial for generating histamine, which can further affect the generation of neurotransmitters in the brain, modulating awareness and addictive behavior, mainly enriched in the frontal cortices [44].

During the late first trimester to second trimester, neurons migrate to the cortical layer and eventually begin to form synapses [69]. In this period, there is a peak in the organization of neuronal populations, laying the groundwork for integrated information processing on a global scale, which is necessary for complex cognitive processes, behavior, and motor functions. In a retrospective study, serum creatinine levels were suggested to predict the risk of adverse pregnancy outcomes, including singleton FGR [75]. The maternal creatinine level not only correlated with fetal muscle content but is also associated with volume deficits in white matter [76], which in turn is associated with motor function in young adults born preterm with a very low birth weight [77].

Previous research has found that school-aged children with singleton FGR exhibit gross and fine motor deficits, cognitive impairments, behavioral dysfunctions, and neurological impairments [78–80]. Recently, Sophie et al. recommended that standardized long-term follow-up for MCDA twins with sFGR is crucial to facilitate early identification of children at risk [66]. In this study, we correlated the levels of differential metabolites with ASQ-3 scores to evaluate the long-term neurocognitive behavioral development of infants and found that l-serine in the first trimester was significantly associated with communication, gross motor function, and problem solving in infants with sFGR. l-arginine in the first trimester is significantly associated with communication, while l-histidine in the first trimester was significantly associated with communication and personal social interactions in sFGR. A decrease in maternal creatinine in the second trimester was correlated with the first walking time and gross motor and fine motor functions in 2- to 3-year-old infants. These results provide further confirmation that changes in metabolites in maternal plasma during pregnancy are closely related to the development of the fetal nervous system.

However, this study has some limitations. First, the ASQ-3 is a screening tool, not a diagnostic tool, for neurodevelopmental delay in children. Indeed, the survey results may not be entirely reliable because the ASQ-3 subscale scores for neurodevelopmental delays in children were obtained from self-report questionnaires completed by the participants. Nevertheless, the ASQ-3 has been validated and is trustworthy [81]. Second, the prediction performance reported for the combinations of metabolites was likely optimistic given that those candidates were selected from a larger pool of metabolites and lacked external validation. Third, the populations in our cohort at each stage are not completely consistent, which may be affected by potential individual heterogeneity. Subsequent studies should expand the sample size and the number of metabolites to assess the repeatability and stability of the results to confirm whether the risk of growth restriction and fetal brain injury in MCDA twins can be evaluated earlier and better. Furthermore, the mechanism that underlies the effect of changes in the maternal plasma metabolite concentration on fetal physical and neurocognitive behavioral development in the first and second trimester remains to be further studied.

## Conclusions

In summary, our results highlight the close relationship between the fluctuation of maternal metabolites in the first and second trimester and both sFGR and adverse neurological outcomes, and these metabolites (l-phenylalanine, l-leucine, and l-isoleucine in the second trimester for sFGR; l-serine, l-arginine, and l-histidine in the first trimester, and creatinine in the second trimester for fetal brain injury) may serve as useful biomarkers to develop strategies for evaluation, prediction, diagnosis, and establishment of promising targets for early intervention with sFGR and adverse neurological outcomes.

### Supplementary Information

**Additional file 1: Table S1.** Fetal physical development parameters estimated by ultrasonography before delivery. **Table S2.** The statistical parameters of the sFGR and each fetal brain injury state. **Table S3.** Reagents and resources. **Table S4.** Metabolite concentrations in maternal plasma during pregnancy quantified by targeted metabolomics. **Table S5.** Metabolite concentrations in cord plasma quantified by targeted metabolomics. **Table S6.** Predictive performance indices of significantly changed metabolites in the prediction of sFGR. **Table S8.** Predictive performance indices of significantly changed metabolites in the prediction of fetal brain injury. **Figure S1.** The detected number of metabolites in maternal plasma and cord plasma. **Figure S2.** The metabolite concentrations of significantly differential metabolites in maternal plasma and cord plasma. **Figure S3.** Maternal metabolite levels in either the sFGR or MCDA group in different trimesters. **Figure S4.** The prediction model of sFGR in maternal plasma and cord plasma. **Figure S5.** Correlation between metabolites in the first trimester and physical development parameters. **Figure S6.** Correlation between metabolites in second and third trimester, and physical development parameters. **Figure S7.** Correlation between metabolites in cord plasma and physical development parameters. **Figure S8.** The differential maternal-fetal metabolite levels and the prediction model of fetal brain injury. **Figure S9.** Correlation between maternal metabolites and first speaking and walking times. **Figure S10.** Summary for the metabolite profile assessment in sensitivity analysis. **Figure S11.** Summary figure of this study.

**Additional file 2: Table S7.** Correlation statistics between metabolite levels and physical development parameters.

**Additional file 3: Table S9.** Metabolite sensitivity analysis results.

## Data Availability

All data generated or analyzed during this study are included in this published article and its additional files.

## Abbreviations

AC: Abdominal circumference
ASQ-3: Ages and Stages Questionnaire-third edition subscale
AUC: Area under the curve
BCAA: Branched-chain amino acids
BMI: Body mass index
BPD: Biparietal diameter
FGR: Fetal growth restriction
FL: Femur length
HC: Head circumference
MCDA: Monochorionic diamniotic
mTOR: Mammalian target of rapamycin
PAGln: Phenylacetylglutamine
PE: Preeclampsia
PLS-DA: Partial least squares discriminant analysis
ROC: Receiver operating characteristic
ROS: Reactive oxygen species
sFGR: Selective fetal growth restriction
TMAO: Trimethylamine-N-oxide
TMVAV: N, N, N-trimethyl-5-aminovaleric acid

## References

[1] Groene SG, Tollenaar LSA, Middeldorp JM, Lopriore E. Neonatal management and outcome in complicated monochorionic twins: what have we learned in the past decade and what should you know? Best Pract Res Clin Obstetr Gynaecol. 2022;84:218–28. 10.1016/j.bpobgyn.2022.03.016 35513960

[2] Lewi L, Deprest J, Hecher K (2013). The vascular anastomoses in monochorionic twin pregnancies and their clinical consequences. Am J Obstet Gynecol.

[3] Buca D, Pagani G, Rizzo G, Familiari A, Flacco ME, Manzoli L, et al. Outcome of monochorionic twin pregnancy with selective intrauterine growth restriction according to umbilical artery Doppler flow pattern of smaller twin: systematic review and meta-analysis. Ultrasound Obstet Gynecol. 2017;50(5):559–68. 10.1002/uog.17362 27859836

[4] Zhu YD, Bian JY, Liao YP, Hu T, Wang MY, Chen YG (2021). Retrospective validation of 11–13 weeks’ gestation ultrasound characteristics as predictive tools for twin-twin transfusion syndrome and selective intrauterine growth restriction in monochorionic diamniotic twin pregnancies. Ann Transl Med.

[5] Gratacós E, Lewi L, Muñoz B, Acosta-Rojas R, Hernandez-Andrade E, Martinez JM (2007). A classification system for selective intrauterine growth restriction in monochorionic pregnancies according to umbilical artery Doppler flow in the smaller twin. Ultrasound Obstet Gynecol.

[6] Bennasar M, Eixarch E, Martinez JM, Gratacós E (2017). Selective intrauterine growth restriction in monochorionic diamniotic twin pregnancies. Semin Fetal Neonatal Med.

[7] GratacÓS E, Eixarch E, Crispi F (2009). Diagnosis and management of selective fetal growth restriction in monochorionic twins. Fetal Mater Med Rev.

[8] Surendran P, Stewart ID, Au Yeung VPW, Pietzner M, Raffler J, Wörheide MA (2022). Rare and common genetic determinants of metabolic individuality and their effects on human health. Nat Med.

[9] Tarca AL, Romero R, Benshalom-Tirosh N, Than NG, Gudicha DW, Done B (2019). The prediction of early preeclampsia: results from a longitudinal proteomics study. PLoS ONE.

[10] Say L, Chou D, Gemmill A, Tunçalp Ö, Moller AB, Daniels J (2014). Global causes of maternal death: a WHO systematic analysis. Lancet Glob Health.

[11] Alkema L, Chou D, Hogan D, Zhang S, Moller AB, Gemmill A (2016). Global, regional, and national levels and trends in maternal mortality between 1990 and 2015, with scenario-based projections to 2030: a systematic analysis by the UN Maternal Mortality Estimation Inter-Agency Group. Lancet (London, England).

[12] Murray E, Fernandes M, Fazel M, Kennedy SH, Villar J, Stein A (2015). Differential effect of intrauterine growth restriction on childhood neurodevelopment: a systematic review. BJOG.

[13] Núñez Estevez KJ, Rondón-Ortiz AN, Nguyen JQT, Kentner AC (2020). Environmental influences on placental programming and offspring outcomes following maternal immune activation. Brain Behav Immun.

[14] Sovio U, Goulding N, McBride N, Cook E, Gaccioli F, Charnock-Jones DS (2020). A maternal serum metabolite ratio predicts fetal growth restriction at term. Nat Med.

[15] Dessì A, Marincola FC, Fanos V (2015). Metabolomics and the great obstetrical syndromes–GDM, PET, and IUGR. Best Pract Res Clin Obstet Gynaecol.

[16] Yang J, Wei Y, Qi H, Yin N, Yang Y, Li Z (2020). Neonatal hair profiling reveals a metabolic phenotype of monochorionic twins with selective intrauterine growth restriction and abnormal umbilical artery flow. Mol Med (Cambridge, Mass).

[17] Wang L, Han TL, Luo X, Li S, Young T, Chen C (2018). Metabolic biomarkers of monochorionic twins complicated with selective intrauterine growth restriction in cord plasma and placental tissue. Sci Rep.

[18] Mitro SD, Wu J, Rahman ML, Cao Y, Zhu Y, Chen Z, et al. Longitudinal plasma metabolomics profile in pregnancy-a study in an ethnically diverse U.S. pregnancy cohort. Nutrients. 2021;13(9):3080. 10.3390/nu13093080 PMC8471130 34578958

[19] Walejko JM, Chelliah A, Keller-Wood M, Wasserfall C, Atkinson M, Gregg A, et al. Diabetes leads to alterations in normal metabolic transitions of pregnancy as revealed by time-course metabolomics. Metabolites. 2020;10(9):350. 10.3390/metabo10090350 PMC7570364 32867274

[20] Ryckman KK, Donovan BM, Fleener DK, Bedell B, Borowski KS (2016). Pregnancy-related changes of amino acid and acylcarnitine concentrations: the impact of obesity. AJP reports.

[21] Lindsay KL, Hellmuth C, Uhl O, Buss C, Wadhwa PD, Koletzko B (2015). Longitudinal metabolomic profiling of amino acids and lipids across healthy pregnancy. PLoS ONE.

[22] Liang L, Rasmussen MH, Piening B, Shen X, Chen S, Röst H (2020). Metabolic dynamics and prediction of gestational age and time to delivery in pregnant women. Cell.

[23] Heath H, Rosario R, McMichael LE, Fanter R, Alarcon N, Quintana-Diaz A (2023). Gestational diabetes is characterized by decreased medium-chain acylcarnitines and elevated purine degradation metabolites across pregnancy: a case-control time-course analysis. J Proteome Res.

[24] Yang J, Wu J, Tekola-Ayele F, Li LJ, Bremer AA, Lu R (2023). Plasma amino acids in early pregnancy and midpregnancy and their interplay with phospholipid fatty acids in association with the risk of gestational diabetes mellitus: results from a longitudinal prospective cohort. Diabetes Care.

[25] Virgiliou C, Gika HG, Witting M, Bletsou AA, Athanasiadis A, Zafrakas M (2017). Amniotic fluid and maternal serum metabolic signatures in the second trimester associated with preterm delivery. J Proteome Res.

[26] Bajoria R, Sooranna SR, Ward S, D’Souza S, Hancock M (2001). Placental transport rather than maternal concentration of amino acids regulates fetal growth in monochorionic twins: Implications for fetal origin hypothesis. Am J Obstet Gynecol.

[27] Yang J, Hou L, Wang J, Xiao L, Zhang J, Yin N (2022). Unfavourable intrauterine environment contributes to abnormal gut microbiome and metabolome in twins. Gut.

[28] Bora SA, Papageorghiou AT, Bottomley C, Kirk E, Bourne T (2008). Reliability of transvaginal ultrasonography at 7–9 weeks’ gestation in the determination of chorionicity and amnionicity in twin pregnancies. Ultrasound Obstet Gynecol.

[29] Menon DK (2005). A retrospective study of the accuracy of sonographic chorionicity determination in twin pregnancies. Twin Res Hum Genet.

[30] Weissmann-Brenner A, Weisz B, Achiron R, Shrim A (2012). Can discordance in CRL at the first trimester predict birth weight discordance in twin pregnancies?. J Perinat Med.

[31] Wan JJ, Schrimmer D, Taché V, Quinn K, Lacoursiere DY, James G (2011). Current practices in determining amnionicity and chorionicity in multiple gestations. Prenat Diagn.

[32] Khalil A, Rodgers M, Baschat A, Bhide A, Gratacos E, Hecher K (2016). ISUOG Practice Guidelines: role of ultrasound in twin pregnancy. Ultrasound Obstet Gynecol.

[33] Squires J, Potter LW, Bricker D (1995). The ASQ User’s Guide for the Ages & Stages Questionnaires: a parent-completed, child-monitoring system.

[34] Kuznetsova A, Brockhoff PB, Rune HB (2017). Christensen: “lmerTest package: tests in linear mixed effects models”. J Stat Software.

[35] Rohart F, Gautier B, Singh A (2017). KA LC: mixOmics: an R package for ‘omics feature selection and multiple data integration. PLoS Comput Biol.

[36] Ripley B, Venables B, Bates DM, Hornik K, Gebhardt A, Firth D (2013). Package ‘mass’. Cran R.

[37] Fox J, Weisberg S, Adler D, Bates D, Baud-Bovy G, Ellison S (2012). Package ‘car’.

[38] Robin X, Turck N, Hainard A, Tiberti N, Lisacek F, Sanchez JC (2011). pROC: an open-source package for R and S+ to analyze and compare ROC curves. BMC Bioinformatics.

[39] Yao M, Yang Z, Rong X, Hu X, Yao N, Zhu M, et al. The exploration of fetal growth restriction based on metabolomics: a systematic review. Metabolites. 2022;12(9):860. 10.3390/metabo12090860 PMC9501562 36144264

[40] Zhao M, Wei H, Li C, Zhan R, Liu C, Gao J (2022). Gut microbiota production of trimethyl-5-aminovaleric acid reduces fatty acid oxidation and accelerates cardiac hypertrophy. Nat Commun.

[41] Zhu W, Gregory JC, Org E, Buffa JA, Gupta N, Wang Z (2016). Gut microbial metabolite TMAO enhances platelet hyperreactivity and thrombosis risk. Cell.

[42] Cosmi E, Visentin S, Favretto D, Tucci M, Ragazzi E, Viel G (2013). Selective intrauterine growth restriction in monochorionic twin pregnancies: markers of endothelial damage and metabolomic profile. Twin Res Hum Genet.

[43] Wallace TC, Blusztajn JK, Caudill MA, Klatt KC, Zeisel SH (2020). Choline: the neurocognitive essential nutrient of interest to obstetricians and gynecologists. J Diet Suppl.

[44] Chabrun F, Dieu X, Rousseau G, Chupin S, Letournel F, Procaccio V (2020). Metabolomics reveals highly regional specificity of cerebral sexual dimorphism in mice. Prog Neurobiol.

[45] Townsend R, D’Antonio F, Sileo FG, Kumbay H, Thilaganathan B, Khalil A (2019). Perinatal outcome of monochorionic twin pregnancy complicated by selective fetal growth restriction according to management: systematic review and meta-analysis. Ultrasound Obstet Gynecol.

[46] Bejar R, Vigliocco G, Gramajo H, Solana C, Benirschke K, Berry C (1990). Antenatal origin of neurologic damage in newborn infantsII Multiple gestations. Am J Obstet Gynecol..

[47] Pharoah POD (2002). Neurological outcome in twins. Seminars Neonatol.

[48] Barker DJ, Osmond C (1986). Infant mortality, childhood nutrition, and ischaemic heart disease in England and Wales. Lancet (London, England).

[49] Barker DJ, Osmond C, Golding J, Kuh D, Wadsworth ME (1989). Growth in utero, blood pressure in childhood and adult life, and mortality from cardiovascular disease. BMJ (Clinical research ed).

[50] Moros G, Boutsikou T, Fotakis C, Iliodromiti Z, Sokou R, Katsila T (2021). Insights into intrauterine growth restriction based on maternal and umbilical cord blood metabolomics. Sci Rep.

[51] Orczyk-Pawilowicz M, Jawien E, Deja S, Hirnle L, Zabek A, Mlynarz P (2016). Metabolomics of human amniotic fluid and maternal plasma during normal pregnancy. PLoS ONE.

[52] Fernstrom JD, Fernstrom MH (2007). Tyrosine, phenylalanine, and catecholamine synthesis and function in the brain. J Nutr..

[53] Muroya S, Zhang Y, Kinoshita A, Otomaru K, Oshima K, Gotoh Y, et al. Maternal undernutrition during pregnancy alters amino acid metabolism and gene expression associated with energy metabolism and angiogenesis in fetal calf muscle. Metabolites. 2021;11(9):582. 10.3390/metabo11090582 PMC8465837 34564398

[54] van den Akker CHP, Schierbeek H, Dorst KY, Schoonderwaldt EM, Vermes A, Duvekot JJ (2009). Human fetal amino acid metabolism at term gestation. Am J Clin Nutr.

[55] Wyant GA, Abu-Remaileh M, Wolfson RL, Chen WW, Freinkman E, Danai LV (2017). mTORC1 activator SLC38A9 is required to efflux essential amino acids from lysosomes and use protein as a nutrient. Cell.

[56] Crozier SJ, Kimball SR, Emmert SW, Anthony JC, Jefferson LS (2005). Oral leucine administration stimulates protein synthesis in rat skeletal muscle. J Nutr.

[57] Nie C, He T, Zhang W, Zhang G, Ma X. Branched chain amino acids: beyond nutrition metabolism. Int J Mol Sci. 2018;19(4):954. 10.3390/ijms19040954 PMC5979320 29570613

[58] Kimball SR, Jefferson LS (2006). New functions for amino acids: effects on gene transcription and translation. Am J Clin Nutr.

[59] Choi W, Kim J, Ko JW, Choi A, Kwon YH (2022). Effects of maternal branched-chain amino acid and alanine supplementation on growth and biomarkers of protein metabolism in dams fed a low-protein diet and their offspring. Amino Acids.

[60] Cetin I, de Santis MS, Taricco E, Radaelli T, Teng C, Ronzoni S (2005). Maternal and fetal amino acid concentrations in normal pregnancies and in pregnancies with gestational diabetes mellitus. Am J Obstet Gynecol.

[61] de Pace V, Chiossi G, Facchinetti F (2007). Clinical use of nitric oxide donors and L-arginine in obstetrics. J Mater Fetal Neonatal Med.

[62] Wang Q, Yue J, Zhou X, Zheng M, Cao B, Li J (2020). Ouabain regulates kidney metabolic profiling in rat offspring of intrauterine growth restriction induced by low-protein diet. Life Sci.

[63] Alexandre-Gouabau MC, Courant F, Le Gall G, Moyon T, Darmaun D, Parnet P (2011). Offspring metabolomic response to maternal protein restriction in a rat model of intrauterine growth restriction (IUGR). J Proteome Res.

[64] Burmester T, Gerlach F, Hankeln T (2007). Regulation and role of neuroglobin and cytoglobin under hypoxia. Adv Exp Med Biol.

[65] Zeisel SH (2011). The supply of choline is important for fetal progenitor cells. Semin Cell Dev Biol.

[66] Groene SG, Stegmeijer KJJ, Tan R, Steggerda SJ, Haak MC, Slaghekke F (2022). Long-term effects of selective fetal growth restriction (LEMON): a cohort study of neurodevelopmental outcome in growth discordant identical twins in the Netherlands. Lancet Child Adolesc Health.

[67] Doi M, Usui N, Shimada S (2022). Prenatal environment and neurodevelopmental disorders. Front Endocrinol.

[68] Stiles J, Jernigan TL (2010). The basics of brain development. Neuropsychol Rev.

[69] Tau GZ, Peterson BS (2010). Normal development of brain circuits. Neuropsychopharmacol.

[70] Paquette AG, Houseman EA, Green BB, Lesseur C, Armstrong DA, Lester B (2016). Regions of variable DNA methylation in human placenta associated with newborn neurobehavior. Epigenetics.

[71] Tachikawa M, Hirose S, Akanuma SI, Matsuyama R, Hosoya KI (2018). Developmental changes of l-arginine transport at the blood-brain barrier in rats. Microvasc Res.

[72] Erens C, Van Broeckhoven J, Bronckaers A, Lemmens S, Hendrix S (2022). The dark side of an essential amino acid: L-arginine in spinal cord injury. J Neurotrauma.

[73] Youssef L, Crovetto F, Simoes RV, Miranda J, Paules C, Blasco M, et al. The interplay between pathophysiological pathways in early-onset severe preeclampsia unveiled by metabolomics. Life (Basel, Switzerland). 2022;12(1):86. 10.3390/life12010086 PMC8780941 35054479

[74] Prokopieva VD, Yarygina EG, Bokhan NA, Ivanova SA (2016). Use of carnosine for oxidative stress reduction in different pathologies. Oxid Med Cell Longev.

[75] Kang J, Hwang S, Lee TS, Cho J, Seo DM, Choi SJ (2022). Gestational age-specific serum creatinine can predict adverse pregnancy outcomes. Sci Rep.

[76] Rajagopalan P, Refsum H, Hua X, Toga AW, Jack CR, Weiner MW (2013). Mapping creatinine- and cystatin C-related white matter brain deficits in the elderly. Neurobiol Aging.

[77] Hollund IMH, Olsen A, Skranes J, Brubakk AM, Håberg AK, Eikenes L (2018). White matter alterations and their associations with motor function in young adults born preterm with very low birth weight. NeuroImage Clin.

[78] Malhotra A, Ditchfield M, Fahey MC, Castillo-Melendez M, Allison BJ, Polglase GR (2017). Detection and assessment of brain injury in the growth-restricted fetus and neonate. Pediatr Res.

[79] Walker DM, Marlow N (2008). Neurocognitive outcome following fetal growth restriction. Arch Dis Child Fetal Neonatal Ed.

[80] Scherjon S, Briët J, Oosting H, Kok J (2000). The discrepancy between maturation of visual-evoked potentials and cognitive outcome at five years in very preterm infants with and without hemodynamic signs of fetal brain-sparing. Pediatrics.

[81] Kobayashi S, Itoh S, Miyashita C, Ait Bamai Y, Yamaguchi T, Masuda H (2022). Impact of prenatal exposure to mercury and selenium on neurodevelopmental delay in children in the Japan environment and Children’s study using the ASQ-3 questionnaire: a prospective birth cohort. Environ Int.

