# Supplementary material for: Metabolic dynamics and prediction of sFGR and adverse fetal outcomes: a prospective longitudinal cohort study
Source: BMC Med. 2023 Nov 23;21:455. doi: 10.1186/s12916-023-03134-9 (PMC10666385; doi:10.1186/s12916-023-03134-9)
Supplement: Supplementary file 1 — Additional file 1: Table S1. Fetal physical development parameters estimated by ultrasonography before delivery. Table S2. The statistical parameters of the sFGR and each fetal brain injury state. Table S3. Reagents and resources. Table S4. Metabolite concentrations in maternal plasma during pregnancy quantified by targeted metabolomics. Table S5. Metabolite concentrations in cord plasma quantified by targeted metabolomics. Table S6. Predictive performance indices of significantly changed metabolites in the prediction of sFGR. Table S8. Predictive performance indices of significantly changed metabolites in the prediction of fetal brain injury. Figure S1. The detected number of metabolites in maternal plasma and cord plasma. Figure S2. The metabolite concentrations of significantly differential metabolites in maternal plasma and cord plasma. Figure S3. Maternal metabolite levels in either the sFGR or MCDA group in different trimesters. Figure S4. The prediction model of sFGR in maternal plasma and cord plasma. Figure S5. Correlation between metabolites in the first trimester and physical development parameters. Figure S6. Correlation between metabolites in second and third trimester, and physical development parameters. Figure S7. Correlation between metabolites in cord plasma and physical development parameters. Figure S8. The differential maternal-fetal metabolite levels and the prediction model of fetal brain injury. Figure S9. Correlation between maternal metabolites and first speaking and walking times. Figure S10. Summary for the metabolite profile assessment in sensitivity analysis. Figure S11. Summary figure of this study. [file 12916_2023_3134_MOESM1_ESM.docx]

**Additional file 1**

**Table S1. Fetal physical development parameters estimated by ultrasonography before delivery**

|  | MCDA (n=84) | sFGR (n=48) | *P* value |
| --- | --- | --- | --- |
| HC (cm) | 29.69 ± 4.12 | 28.34 ± 2.73 | <0.001 |
| AC (cm) | 28.34 ± 4.29 | 25.83 ± 4.03 | <0.001 |
| BPD (cm) | 8.21 ± 1.21 | 7.87 ± 0.87 | <0.001 |
| FL (cm) | 6.24 ± 1.01 | 5.71 ± 0.82 | <0.001 |
| HC/AC ratio | 1.06 ± 0.06 | 1.11 ± 0.09 | 0.001 |
| FL/BPD ratio | 0.76 ± 0.05 | 0.72 ± 0.05 | <0.001 |
| FL/AC ratio | 0.22 ± 0.01 | 0.22 ± 0.01 | 0.825 |

HC: head circumference; AC: abdominal circumference; BPD: biparietal diameter; FL: femur length; MCDA: normal monochorionic diamniotic twins; sFGR: selective fetal growth restriction; n: represents the number of fetuses in the group; Data were analysed by Mann‒Whitney U test; Continuous variables were presented as the mean ± standard deviation (SD); *P*<0.05 was considered statistically significant.

**Table S2 The statistical parameters of the sFGR and each fetal brain injury state.**

|  | MCDA (n=88) | sFGR (n=68) | Estimate | z value | Pr (>\|z\|) |
| --- | --- | --- | --- | --- | --- |
| Abnormal brain structure | 15 | 24 | 2.22 | 1.87 | 0.062 |
| Abnormal choroid plexus | 6 | 14 | 1.60 | 2.06 | 0.040 |
| White matter damage | 8 | 19 | 2.14 | 1.47 | 0.141 |

MCDA: normal monochorionic diamniotic twins; sFGR: selective fetal growth restriction; n: represents the number of fetuses in the group; Estimate: represents the estimated coefficient for sFGR in the model. z value: represents a statistical measure used to assess the significance of the coefficient estimate; Pr(>|z|): represents the P value associated with the z value; Data were presented as number; Mixed effects logistic regression model were used to evaluate the statistical correlation between sFGR and fetal brain injury; *P*<0.05 was considered statistically significant.

**Table S3 Reagents and resources**

| Chemicals | Source | Identifier |
| --- | --- | --- |
| Amino Acid Standard (AccQ-Tag,Pico-Tag,,AccQ-Tag Ultra) | Waters | WAT088122 |
| Cell free amino acid mixture-13C,15N, 98 atom % 13C, 98 atom % 15N | Aldrich | 767964 |
| Trimethylamine N-oxide | Aldrich | 317594 |
| Choline chloride | Sigma | C7017 |
| Betaine | Sigma | B2629 |
| (3-Carboxypropyl) trimethylammonium chloride | Aldrich | 403245 |
| L-Carnitine | cayman | 21489 |
| Creatinine | Sigma-Aldrich | C4255 |
| Nε,Nε,Nε-Trimethyllysine hydrochloride | Sigma | T1660 |
| N, N-Dimethylglycine | Sigma | D1156 |
| TRIMETHYLAMINE N-OXIDE (D9, 98%) | CIL | DLM-4779-1 |
| CHOLINE CHLORIDE (TRIMETHYL-D9, 98%) | CIL | DLM-549-1 |
| BETAINE (D11, 98%) | CIL | DLM-407-1 |
| N- (Carboxypropyl)-N, N, N-trimethyl-d9-ammonium Chloride | CDN | D-6789 |
| L-CARNITINE (TRIMETHYL-D9, 98%) | CDN | DLM-3555-5 |
| Nε, Nε, Nε-Trimethyllysine-d9 | TRC | T796447 |
| Phenylacetylglutamine (PAGln) | Santa Cruz Biotechnology | SC-212551A |
| D5-Phenylacetylglutamine (D5-PAgln) | CDN Isotopes | D-6900 |
| L-13C9-15N1-Phenylalaine | Aldrich | 608017 |
| L-Phenylalanine | Sigma | P5482 |
| Hippuric acid | Sigma-Aldrich | 68069 |
| Acetonitrile UHPLC-MS | Thermo Fisher Scientific | A956-1 |
| Isopropanol Optima LC-MS | Fisher Chemicals | A461-212 |
| Water UHPLC-MS | Thermo Fisher Scientific | W8-1 |
| Methanol Optima LC-MS | Fisher Chemicals | A454-4 |
| Ammonium formate Optima LC-MS | Fisher Chemicals | A115-50 |
| Ammonium acetate Optima LC-MS | Fisher Chemicals | A114-50 |
| Formic acid Optima LC-MS | Fisher Chemicals | A117-50 |
| Acetic acid Optima LC-MS | Fisher Chemicals | A113-50 |
| LC-20AD Shimadazu pump system | Shimadzu Scientific Instruments | N/A |
| Triple Quad 6500+ LC-MS/MS | Sciex | N/A |
| Luna® 5 µm Silica (2) 100 Å, LC Column 100 x 2 mm | Phenomenex | 00D-4274-B0 |
| Kinetex C18 column (50 mm 3 2.1 mm 2.6 mm) | Phenomenex | 00B-4462-AN |
| Intrada Amino Acid, 100 x 3 mm | Imtakt | TI770E-NJ06 |

**Table S4. Metabolite concentrations in maternal plasma during pregnancy quantified by targeted metabolomics.**

|  | FT | | |  | ST | | |  | TT | | |
| --- | --- | --- | --- | --- | --- | --- | --- | --- | --- | --- | --- |
|  | MCDA (n=41) | sFGR (n=13) | *P* value |  | MCDA (n=43) | sFGR (n=23) | *P* value |  | MCDA (n=43) | sFGR (n=23) | *P* value |
| L-alanine | 572.00 (522.50-677.00) | 588.00 (484.00-642.50) | 0.32 ^a^ |  | 518.00 (431.00-618.00 | 467.00 (408.00-548.00) | 0.053 ^a^ |  | 569.00 (473.00-634.00) | 603.00 (527.00-679.00) | 0.27 ^b^ |
| L-serine | 30.75 (21.73-38.43) | 21.90 (15.70-30.50) | 0.10 ^b^ |  | 28.80 (18.80-37.9 | 30.40 (22.10-47.80) | 0.56 ^a^ |  | 50.8 (30.90-63.00) | 47.10 (33.80-61.20) | 0.88 ^a^ |
| L-valine | 244.00 (182.50-265.50) | 213.00 (158.50-232.50) | 0.38 ^a^ |  | 265.00 (244.00-285.00) | 242.00 (208.00-292.00) | 0.07 ^a^ |  | 212.00 (194.00-247.00) | 219.00 (168.00-264.00) | 0.70 ^b^ |
| L-threonine | 142.00 (122.50-173.00) | 155.00 (131.50-181.00) | 0.77 ^b^ |  | 199.00 (173.00-229.00) | 193.00 (159.00-207.00) | 0.15 ^a^ |  | 183.00 (162.00-223.00) | 209.00 (163.00-254.00) | 0.17 ^b^ |
| L-lysine | 210.00 (188.25-230.75) | 194.00 (175.50-212.00) | 0.07 ^a^ |  | 202.00 (178.00-227.00) | 192.00 (163.00-229.00) | 0.28 ^a^ |  | 184.00 (149.00-214.00) | 182.00 (144.00-221.00) | 0.99 ^b^ |
| L-methionine | 45.10 (36.10-56.10) | 40.30 (35.95-46.65) | 0.27 ^b^ |  | 30.80 (25.30-42.70) | 25.00 (18.70-41.40) | 0.08 ^b^ |  | 26.30 (20.00-31.10) | 28.70 (17.60-37.40) | 0.83 ^a^ |
| L-cystine | 78.70 (53.85-140.00) | 61.50 (33.10-83.75) | 0.17 ^b^ |  | 81.50 (40.80-124.00) | 49.30 (29.30-96.70) | 0.12 ^b^ |  | 58.60 (35.50-79.30) | 52.30 (34.40-95.90) | 0.96 ^b^ |
| carinitine | 114.00 (73.10-136.50) | 101.00 (62.80-157.00) | 0.53 ^a^ |  | 87.40 (74.20-97.00) | 81.90 (76.10-97.40) | 0.83 a |  | 60.10 (52.70-71.30) | 64.30 (53.45-73.10) | 0.68 ^a^ |
| TMAO | 0.21 (0.09-0.39) | 0.10 (0.09-0.30) | 0.38 ^b^ |  | 0.41 (0.20-0.61) | 0.41 (0.31-0.64) | 0.30 ^b^ |  | 0.40 (0.24-0.67) | 0.33 (0.20-0.59) | 0.39 ^b^ |
| choline | 6.46 (3.97-8.26) | 7.87 (4.50-9.56) | 0.47 ^b^ |  | 11.40 (10.10-12.80) | 12.00 (10.90-13.70) | 0.26 ^b^ |  | 10.50 (9.03-12.80) | 12.10 (9.37-14.53) | 0.14 ^a^ |
| butyrobetaine | 0.67 (0.36-0.88) | 0.52 (0.31-0.78) | 0.41 ^b^ |  | 0.71 (0.60-0.78) | 0.66 (0.62-0.75) | 0.80 ^b^ |  | 0.54 (0.48-0.61) | 0.55 (0.49-0.69) | 0.55 ^b^ |
| trimethyllysine | 0.49 (0.28-0.60) | 0.46 (0.27-0.80) | 0.64 ^a^ |  | 0.72 (0.62-0.84) | 0.75 (0.65-0.79) | 0.70 ^b^ |  | 0.67 (0.55-0.75) | 0.67 (0.59-0.88) | 0.49 ^b^ |
| creatintine | 21.90 (16.60-30.45) | 22.30 (17.30-41.80) | 0.28 ^b^ |  | 38.80 (29.70-53.30) | 35.10 (27.70-44.00) | 0.46 ^b^ |  | 36.30 (25.00-44.20) | 33.80 (25.88-44.73) | 0.83 ^b^ |
| hippuric acid | 0.54 (0.25-1.37) | 0.60 (0.16-0.86) | 0.41 ^b^ |  | 2.78 (1.34-4.70) | 1.74 (0.94-3.75) | 0.11 ^b^ |  | 1.23 (0.57-2.18) | 1.03 (0.37-1.38) | 0.39 ^b^ |
| PAGln | 3.32 (2.31-4.39) | 3.25 (2.10-4.90) | 1.00 ^b^ |  | 1.81 (1.23-2.39) | 1.53 (0.99-2.87) | 0.99 ^b^ |  | 2.34 (1.70-3.72) | 2.21 (1.46-3.64) | 0.76 ^b^ |

FT: first trimester; ST: second trimester; TT: third trimester; MCDA: normal monochorionic diamniotic twins; sFGR: selective fetal growth restriction; TMAO: trimethylamine-N-oxide; PAGln: phenylacetylglutamine; n: represents the number of pregnant women in the group; n: represents the number of fetuses in the group; ^a^ Student’s t-test, ^b^ Mann‒Whitney U test; Data were presented as median and interquartile range (25%-75%) of measured concentrations (μM). *P*<0.05 was considered statistically significant.

**Table S5 Metabolite concentrations in cord plasma quantified by targeted metabolomics**

|  | MCDA (n=85) | sFGR (n=63) | *P* value | sFGR-L (n=33) | sFGR-S (n=32) | *P* value |
| --- | --- | --- | --- | --- | --- | --- |
| L-alanine | 541.00 (418.00-695.00) | 550.00 (479.00-698.50) | 0.26 ^b^ | 540.00 (479.00-629.00) | 611.00 (471.50-733.50) | 0.45 ^d^ |
| L-serine | 143.00 (120.50-175.50) | 140.00 (113.50-175.50) | 0.73 ^b^ | 138.00 (113.50-183.50) | 140.50 (111.25-171.50) | 0.22 ^d^ |
| L-proline | 245.00 (205.00-285.00) | 242.00 (211.00-320.50) | 0.40 ^b^ | 231.00 (208.50-302.50) | 256.50 (222.50-339.25) | 0.07 ^d^ |
| L-valine | 273.50 (230.25-320.75) | 301.00 (262.50-362.00) | 0.005 ^b^ | 291.00 (262.50-356.00) | 309.50 (262.75-365.75) | 0.65 ^d^ |
| L-threonine | 311.00 (257.25-406.25) | 382.00 (303.50-471.00) | 0.004 ^a^ | 348.00 (297.00-458.50) | 403.50 (315.75-477.25) | 0.69 ^c^ |
| L-lysine | 363.50 (284.50-429.50) | 391.00 (325.00-467.00) | 0.41 ^a^ | 372.00 (322.00-453.50) | 404.00 (325.00-479.00) | 0.64 ^c^ |
| L-glutamic acid | 357.50 (222.00-488.25) | 323.00 (191.50-500.00) | 0.57 ^b^ | 378.00 (254.00-508.50) | 276.00 (172.25-435.75) | 0.34 ^d^ |
| L-methionine | 42.50 (32.83-60.53) | 51.50 (34.65-62.75) | 0.53 ^a^ | 51.90 (36.45-62.15) | 48.30 (30.53-64.35) | 0.47 ^c^ |
| L-histidine | 157.00 (129.50-180.75) | 164.00 (131.50-192.00) | 0.24 ^a^ | 153.00 (132.00-178.00) | 171.50 (129.75-216.25) | 0.22 ^c^ |
| L-phenylalanine | 91.25 (78.03-111.75) | 96.20 (80.65-108.00) | 0.62 ^b^ | 95.30 (88.60-108.00) | 96.60 (74.60-111.50) | 0.54 ^d^ |
| L-arginine | 52.15 (29.33-66.50) | 59.80 (40.55-86.75) | 0.06 ^a^ | 58.90 (39.90-86.75) | 59.95 (40.45-87.90) | 0.90 ^d^ |
| L-tyrosine | 69.60 (60.90-83.73) | 78.80 (63.55-94.30) | 0.07 ^b^ | 78.00 (65.10-91.20) | 80.15 (61.30-97.25) | 0.72 ^d^ |
| L-cystine | 54.90 (34.35-106.50) | 52.60 (26.05-112.00) | 0.52 ^b^ | 52.60 (27.90-105.50) | 53.10 (16.40-116.00) | 0.52 ^d^ |
| L-leucine | 117.00 (87.28-151.00) | 135.00 (101.50-170.50) | 0.01 ^b^ | 123.00 (101.50-165.00) | 149.00 (102.50-175.00) | 0.66 ^d^ |
| L-isoleucine | 74.05 (53.10-86.00) | 81.60 (66.40-99.95) | 0.004 ^a^ | 82.80 (66.40-93.90) | 80.50 (64.03-103.75) | 0.82 ^c^ |
| carinitine | 49.00 (35.25-60.95) | 51.00 (40.30-71.60) | 0.27 ^b^ | 49.60 (40.60-67.05) | 53.95 (37.63-76.13) | 0.97 ^d^ |
| TMAO | 0.51 (0.26-0.91) | 0.63 (0.31-1.09) | 0.25 ^b^ | 0.54 (0.31-1.02) | 0.79 (0.25-1.18) | 0.73 ^d^ |
| betaine | 36.20 (28.75-45.40) | 34.30 (28.40-40.60) | 0.25 ^a^ | 33.10 (27.75-39.90) | 34.75 (30.23-45.08) | 0.20 ^c^ |
| choline | 30.30 (25.70-34.80) | 34.30 (27.70-41.00) | 0.02 ^b^ | 30.80 (26.65-40.30) | 35.65 (30.55-42.73) | 0.73 ^d^ |
| butyrobetaine | 0.55 (0.35-0.84) | 0.57 (0.39-0.89) | 0.59 ^b^ | 0.46 (0.37-0.88) | 0.64 (0.40-0.89) | 0.45 ^d^ |
| trimethyllysine | 1.06 (0.80-1.40) | 1.13 (0.86-1.64) | 0.22 ^b^ | 1.03 (0.88-1.61) | 1.14 (0.84-1.66) | 0.95 ^d^ |
| TMVAV | 0.58 (0.47-0.78) | 0.63 (0.51-0.77) | 0.47 ^b^ | 0.66 (0.51-0.79) | 0.61 (0.51-0.74) | 0.64 ^d^ |
| creatintine | 50.80 (44.45-63.65) | 55.30 (46.10-65.50) | 0.55 ^b^ | 55.00 (44.30-61.80) | 56.00 (49.25-65.73) | 0.66 ^d^ |
| hippuric acid | 1.18 (0.63-1.53) | 1.48 (0.78-2.60) | 0.01 ^b^ | 1.47 (0.83-2.37) | 1.49 (0.75-2.66) | 0.72 ^d^ |
| PAGln | 2.77 (1.89-3.98) | 2.97 (1.82-4.55) | 0.78 ^b^ | 3.18 (1.59-4.61) | 2.74 (1.93-4.62) | 0.61 ^d^ |

MCDA: normal monochorionic diamniotic twins; sFGR: selective fetal growth restriction; sFGR-L: the larger fetus in selective fetal growth restriction; sFGR-S: the smaller fetus in selective fetal growth restriction; TMAO: trimethylamine-N-oxide; TMVAV: N, N, N-trimethyl-5-aminovaleric acid; PAGln: phenylacetylglutamine; ^a^ Student’s t-test, ^b^ Mann‒Whitney U test; ^c^ Paired t-test; ^d^ Wilcoxon's-sign-rank-test. Data are presented as the median and interquartile range (25%-75%) of measured concentrations (μM). *P*<0.05 was considered statistically significant.

**Table S6 Predictive performance indices of significantly changed metabolites in the prediction of sFGR.**

| Metabolites | AUC (95% CI) | *P* value | Best Cutoff | sensitivity | specificity | Youden index | +Likelihood Ratio | -Likelihood Ratio | Accuracy | 90% Cutoff | sensitivity | specificity | Youden index | +Likelihood Ratio | -Likelihood Ratio | Accuracy |
| --- | --- | --- | --- | --- | --- | --- | --- | --- | --- | --- | --- | --- | --- | --- | --- | --- |
| FT-L-leucine | 0.79 (0.65-0.93) | <0.001 | 0.21 | 0.92 | 0.71 | 0.62 | 3.13 | 0.12 | 0.76 | 0.40 | 0.33 | 0.90 | 0.24 | 3.42 | 0.74 | 0.77 |
| ST-L-proline | 0.71 (0.57-0.85) | 0.004 | 0.41 | 0.59 | 0.84 | 0.43 | 3.63 | 0.49 | 0.75 | 0.50 | 0.27 | 0.91 | 0.18 | 2.93 | 0.80 | 0.69 |
| ST-L-phenylalanine | 0.79 (0.67-0.90) | <0.001 | 0.29 | 0.86 | 0.65 | 0.52 | 2.48 | 0.21 | 0.72 | 0.55 | 0.36 | 0.91 | 0.27 | 3.91 | 0.70 | 0.72 |
| ST-L-arginine | 0.69 (0.55-0.83) | 0.008 | 0.33 | 0.73 | 0.67 | 0.40 | 2.23 | 0.40 | 0.69 | 0.47 | 0.23 | 0.91 | 0.13 | 2.44 | 0.85 | 0.68 |
| ST-L-tyrosine | 0.73 (0.61-0.86) | <0.001 | 0.41 | 0.59 | 0.81 | 0.41 | 3.18 | 0.50 | 0.74 | 0.56 | 0.32 | 0.91 | 0.23 | 3.42 | 0.75 | 0.71 |
| ST-L-leucine | 0.72 (0.59-0.86) | 0.001 | 0.35 | 0.73 | 0.70 | 0.43 | 2.41 | 0.39 | 0.71 | 0.50 | 0.36 | 0.91 | 0.27 | 3.91 | 0.70 | 0.72 |
| ST-L-isoleucine | 0.65 (0.49-0.82) | 0.070 | 0.38 | 0.64 | 0.81 | 0.45 | 3.42 | 0.45 | 0.75 | 0.42 | 0.36 | 0.91 | 0.27 | 3.91 | 0.70 | 0.72 |
| ST-betaine | 0.65 (0.51-0.80) | 0.039 | 0.35 | 0.68 | 0.67 | 0.36 | 2.09 | 0.47 | 0.68 | 0.49 | 0.09 | 0.91 | -0.002 | 0.98 | 1.00 | 0.63 |
| ST-TMVAV | 0.66 (0.53-0.79) | 0.017 | 0.32 | 0.77 | 0.54 | 0.31 | 1.66 | 0.42 | 0.62 | 0.48 | 0.09 | 0.91 | -0.002 | 0.98 | 1.00 | 0.63 |
| **ST-Model A-eight** | 0.89 (0.81-0.96) | <0.001 | 0.18 | 0.96 | 0.67 | 0.63 | 2.93 | 0.07 | 0.77 | 0.57 | 0.59 | 0.91 | 0.50 | 6.35 | 0.45 | 0.80 |
| **ST-Model B-three** | 0.88 (0.80-0.96) | <0.001 | 0.33 | 0.82 | 0.77 | 0.59 | 3.52 | 0.24 | 0.79 | 0.52 | 0.64 | 0.91 | 0.54 | 6.84 | 0.40 | 0.82 |
| TT-L-glutamic acid | 0.69 (0.54-0.83) | 0.010 | 0.30 | 0.77 | 0.58 | 0.35 | 1.85 | 0.39 | 0.65 | 0.50 | 0.36 | 0.91 | 0.27 | 3.91 | 0.70 | 0.72 |
| TT-L-histidine | 0.73 (0.59-0.87) | 0.001 | 0.50 | 0.46 | 0.93 | 0.39 | 6.52 | 0.59 | 0.77 | 0.48 | 0.46 | 0.91 | 0.36 | 4.89 | 0.60 | 0.75 |
| **TT-Model A-Two** | 0.74 (0.61-0.88) | 0.001 | 0.40 | 0.68 | 0.81 | 0.50 | 3.66 | 0.39 | 0.77 | 0.48 | 0.41 | 0.91 | 0.32 | 4.40 | 0.65 | 0.74 |
| CP-L-valine | 0.64 (0.55-0.73) | 0.002 | 0.50 | 0.43 | 0.82 | 0.24 | 2.30 | 0.70 | 0.65 | 0.60 | 0.13 | 0.90 | 0.03 | 1.33 | 0.96 | 0.57 |
| CP-L-threonine | 0.65 (0.55-0.74) | 0.002 | 0.49 | 0.49 | 0.74 | 0.23 | 1.90 | 0.69 | 0.63 | 0.56 | 0.30 | 0.90 | 0.20 | 2.99 | 0.78 | 0.64 |
| CP-L-isoleucine | 0.63 (0.54-0.72) | 0.005 | 0.46 | 0.57 | 0.67 | 0.24 | 1.72 | 0.64 | 0.63 | 0.57 | 0.18 | 0.91 | 0.09 | 2.09 | 0.90 | 0.60 |
| CP-L-leucine | 0.62 (0.52-0.71) | 0.010 | 0.36 | 0.85 | 0.35 | 0.20 | 1.30 | 0.43 | 0.56 | 0.53 | 0.23 | 0.90 | 0.13 | 2.32 | 0.86 | 0.61 |
| CP-choline | 0.62 (0.53-0.72) | 0.010 | 0.44 | 0.56 | 0.73 | 0.29 | 2.05 | 0.61 | 0.66 | 0.48 | 0.23 | 0.90 | 0.13 | 2.32 | 0.86 | 0.61 |
| CP-hippuric acid | 0.63 (0.54-0.73) | 0.007 | 0.46 | 0.49 | 0.78 | 0.27 | 2.21 | 0.65 | 0.66 | 0.53 | 0.28 | 0.90 | 0.18 | 2.82 | 0.80 | 0.63 |
| **CP-Model A-six** | 0.72 (0.63-0.80) | <0.001 | 0.51 | 0.56 | 0.82 | 0.37 | 3.01 | 0.54 | 0.70 | 0.59 | 0.38 | 0.90 | 0.28 | 3.82 | 0.69 | 0.68 |
| **CP-Model B-three** | 0.70 (0.62-0.79) | <0.001 | 0.55 | 0.43 | 0.89 | 0.32 | 3.84 | 0.65 | 0.69 | 0.59 | 0.34 | 0.90 | 0.25 | 3.49 | 0.73 | 0.66 |

**ST-Model A-eight:** LOGIT (P (sFGR)) = 51.528 -1.076* `ST-L-proline` -8.374 *`ST-L-phenylalanine` +0.618* `ST-L-arginine`+0.379*`ST-L-tyrosine`-4.444 *`ST-L-leucine`+5.874*`ST-L-isoleucine` -0.374 *ST-betaine +1.150 * ST-TMVAV; **ST-Model B-three:** LOGIT (P (sFGR)) = 47.26-7.85 *`ST-L-phenylalanine`-4.23*`ST-L-leucine`+5.18*`ST-L-isoleucine`; **TT-Model A-Two:** LOGIT (P (sFGR))=21.211+0.803*`TT-L-glutamic acid`+2.025*`TT-L-histidine`; **CP-Model A-six:** LOGIT (P (sFGR)) = -20.0414+0.9512* `L-valine`+0.5631* `L-threonine`+0.7490*`L-isoleucine`-0.0891*`L-leucine`+0.3922*choline+0.9155*`hippuric acid`; **CP-Model B-three:** LOGIT (P (sFGR))=-15.586+1.049* `L-valine`+ 0.875*`L-isoleucine`+0.954*`hippuric acid`; AUC: area under curve; CI: confidence interval; sFGR: selective fetal growth restriction; TMVAV: N, N, N-trimethyl-5-aminovaleric acid; FT: first trimester, ST: second trimester, TT: third trimester; CP: cord plasma; The index in each model were obtained after selecting the cut-off based on the Youden index.

**Table S8 Predictive performance indices of significantly changed metabolites in the prediction of fetal brain injury.**

| Metabolites | AUC (95% CI) | *P* value | Best Cutoff | sensitivity | specificity | Youden index | +Likelihood Ratio | -Likelihood Ratio | Accuracy | 90% Cutoff | sensitivity | specificity | Youden index | +Likelihood Ratio | -Likelihood Ratio | Accuracy |
| --- | --- | --- | --- | --- | --- | --- | --- | --- | --- | --- | --- | --- | --- | --- | --- | --- |
| CP-L-arginine | 0.63 (0.53-0.74) | 0.009 | 0.27 | 0.63 | 0.68 | 0.31 | 1.96 | 0.55 | 0.67 | 0.35 | 0.06 | 0.91 | -0.03 | 0.62 | 1.04 | 0.70 |
| CP-creatintine | 0.61 (0.51-0.72) | 0.04 | 0.283 | 0.429 | 0.789 | 0.218 | 2.03 | 0.724 | 0.701 | 0.329 | 0.2 | 0.908 | 0.1083 | 2.18 | 0.881 | 0.736 |
| **CP-Model A-two** | 0.69 (0.59-0.79) | <0.001 | 0.229 | 0.771 | 0.569 | 0.34 | 1.79 | 0.402 | 0.618 | 0.389 | 0.2286 | 0.908 | 0.1368 | 2.491 | 0.849 | 0.743 |
| FT-L-serine | 0.70 (0.54-0.87) | 0.02 | 0.237 | 0.786 | 0.583 | 0.369 | 1.89 | 0.367 | 0.64 | 0.473 | 0.2857 | 0.917 | 0.2024 | 3.429 | 0.779 | 0.74 |
| FT-L-histidine | 0.69 (0.51-0.87) | 0.03 | 0.349 | 0.571 | 0.833 | 0.405 | 3.43 | 0.514 | 0.76 | 0.446 | 0.3571 | 0.917 | 0.2738 | 4.286 | 0.701 | 0.76 |
| FT-L-arginine | 0.72 (0.58-0.87) | 0.003 | 0.244 | 0.857 | 0.583 | 0.44 | 2.06 | 0.245 | 0.66 | 0.532 | 0.0714 | 0.917 | -0.0119 | 0.857 | 1.013 | 0.68 |
| ST-creatintine | 0.67 (0.51-0.83) | 0.04 | 0.28 | 0.714 | 0.583 | 0.298 | 1.71 | 0.49 | 0.62 | 0.414 | 0.2857 | 0.917 | 0.2024 | 3.429 | 0.779 | 0.74 |
| TT-L-glutamic acid | 0.66 (0.49-0.83) | 0.06 | 0.206 | 0.929 | 0.389 | 0.317 | 1.52 | 0.184 | 0.54 | 0.452 | 0.2143 | 0.917 | 0.131 | 2.571 | 0.857 | 0.72 |
| **MP-Model A-five** | 0.94 (0.88-1.00) | <0.001 | 0.119 | 1 | 0.75 | 0.75 | 4 | 0 | 0.82 | 0.426 | 0.7857 | 0.917 | 0.7024 | 9.429 | 0.234 | 0.88 |
| **MP-Model A-four** | 0.94 (0.88-1.00) | <0.001 | 0.119 | 1 | 0.75 | 0.75 | 4 | 0 | 0.82 | 0.418 | 0.7857 | 0.917 | 0.7024 | 9.429 | 0.234 | 0.88 |
| ST-L-proline | 0.58 (0.43-0.73) | 0.32 | 0.309 | 0.762 | 0.419 | 0.181 | 1.31 | 0.569 | 0.531 | 0.408 | 0.1905 | 0.907 | 0.0975 | 2.05 | 0.893 | 0.672 |
| ST-L-phenylalanine | 0.62 (0.47-0.78) | 0.11 | 0.336 | 0.619 | 0.628 | 0.247 | 1.66 | 0.607 | 0.625 | 0.421 | 0.2857 | 0.907 | 0.1927 | 3.07 | 0.788 | 0.703 |
| ST-L-arginine | 0.61 (0.46-0.76) | 0.14 | 0.367 | 0.429 | 0.791 | 0.219 | 2.05 | 0.723 | 0.672 | 0.437 | 0.1905 | 0.907 | 0.0975 | 2.05 | 0.893 | 0.672 |
| ST-L-tyrosine | 0.58 (0.42-0.74) | 0.33 | 0.329 | 0.571 | 0.674 | 0.246 | 1.76 | 0.635 | 0.641 | 0.41 | 0.2381 | 0.907 | 0.1451 | 2.56 | 0.84 | 0.688 |
| ST-L-leucine | 0.50 (0.33-0.66) | 0.95 | 0.36 | 0.143 | 1 | 0.143 | Inf | 0.857 | 0.719 | 0.343 | 0.1429 | 0.907 | 0.0498 | 1.54 | 0.945 | 0.656 |
| ST-L-isoleucine | 0.64 (0.49-0.80) | 0.07 | 0.311 | 0.714 | 0.581 | 0.296 | 1.71 | 0.491 | 0.625 | 0.431 | 0.2857 | 0.907 | 0.1927 | 3.07 | 0.788 | 0.703 |
| ST-betaine | 0.52 (0.37-0.68) | 0.76 | 0.289 | 1 | 0.14 | 0.14 | 1.16 | 0 | 0.422 | 0.359 | 0.1429 | 0.907 | 0.0498 | 1.54 | 0.945 | 0.656 |
| ST-TMVAV | 0.52 (0.37-0.67) | 0.78 | 0.339 | 0.476 | 0.674 | 0.151 | 1.46 | 0.777 | 0.609 | 0.365 | 0.0952 | 0.93 | 0.0255 | 1.37 | 0.973 | 0.656 |
| **MP-ST-Model A-eight** | 0.76 (0.64-0.88) | <0.001 | 0.323 | 0.762 | 0.721 | 0.483 | 2.73 | 0.33 | 0.734 | 0.59 | 0.2857 | 0.907 | 0.1927 | 3.07 | 0.788 | 0.703 |
| **MP-ST-Model A-two** | 0.73 (0.60-0.86) | <0.001 | 0.24 | 0.91 | 0.54 | 0.44 | 1.95 | 0.18 | 0.66 | 0.52 | 0.38 | 0.91 | 0.29 | 4.10 | 0.68 | 0.73 |

**CP-Model A-two:** LOGIT (P (fetal brain injury)) = 2.711 +0.647*L-arginine -1.321*creatintine; **MP-Model A-five:** LOGIT (P (fetal brain injury)) = 17.515-1.693*`FT-L-serine`-5.091 *`FT-L-histidine`+6.697*`FT-L-arginine`-2.293*`ST-creatintine`+ 0.098*`TT-L-glutamic acid`; **MP-Model B-four:** LOGIT (P (fetal brain injury)) = 18.66-1.72*`FT-L-serine`-5.18*`FT-L-histidine`+ 6.75*`FT-L-arginine`-2.29*`ST-creatintine`; **MP-ST-Model A-eight:** LOGIT (P (fetal brain injury))=17.858-0.844*`ST-L-proline`-4.455*`ST-L-phenylalanine`+1.497*`ST-L-arginine`+3.899*`ST-L-tyrosine`+1.046*`ST-L-leucine`-3.450*`ST-L-isoleucine`-0.418*`ST-betaine`-1.645*`ST-TMVAV`; **MP-ST-Model B-two:** LOGIT (P (fetal brain injury))=9.95-3.10*`ST-L-phenylalanine`+1.61*`ST-L-arginine`; AUC: area under curve; CI: confidence interval; TMVAV: N, N, N-trimethyl-5-aminovaleric acid; FT: first trimester, ST: second trimester, TT: third trimester; CP: cord plasma; Inf: infinity; The index in each model were obtained after selecting the cut-off based on the Youden index.


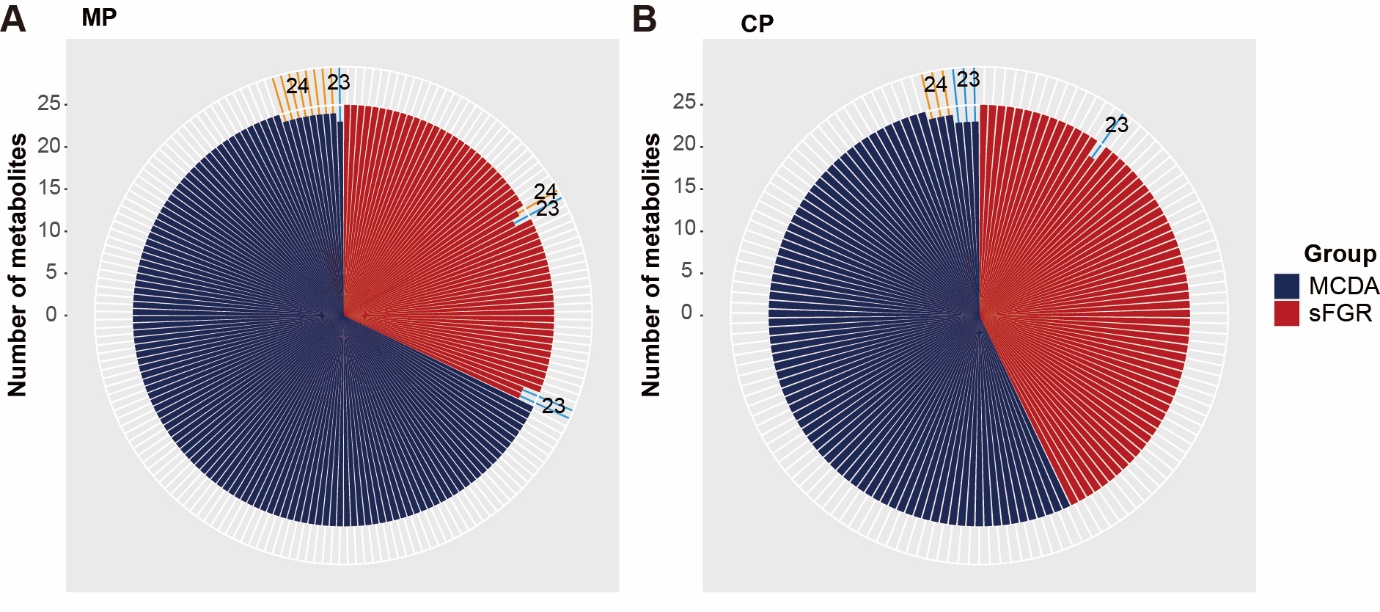


**Figure S1 The detected number of metabolites in maternal plasma and cord plasma.**

A, The rose map showing the number of metabolites detected in maternal plasma for each individual in a special trimester; B, The rose map showing the number of metabolites detected in cord blood plasma for each individual; MP: maternal plasma; CP: cord plasma;

**
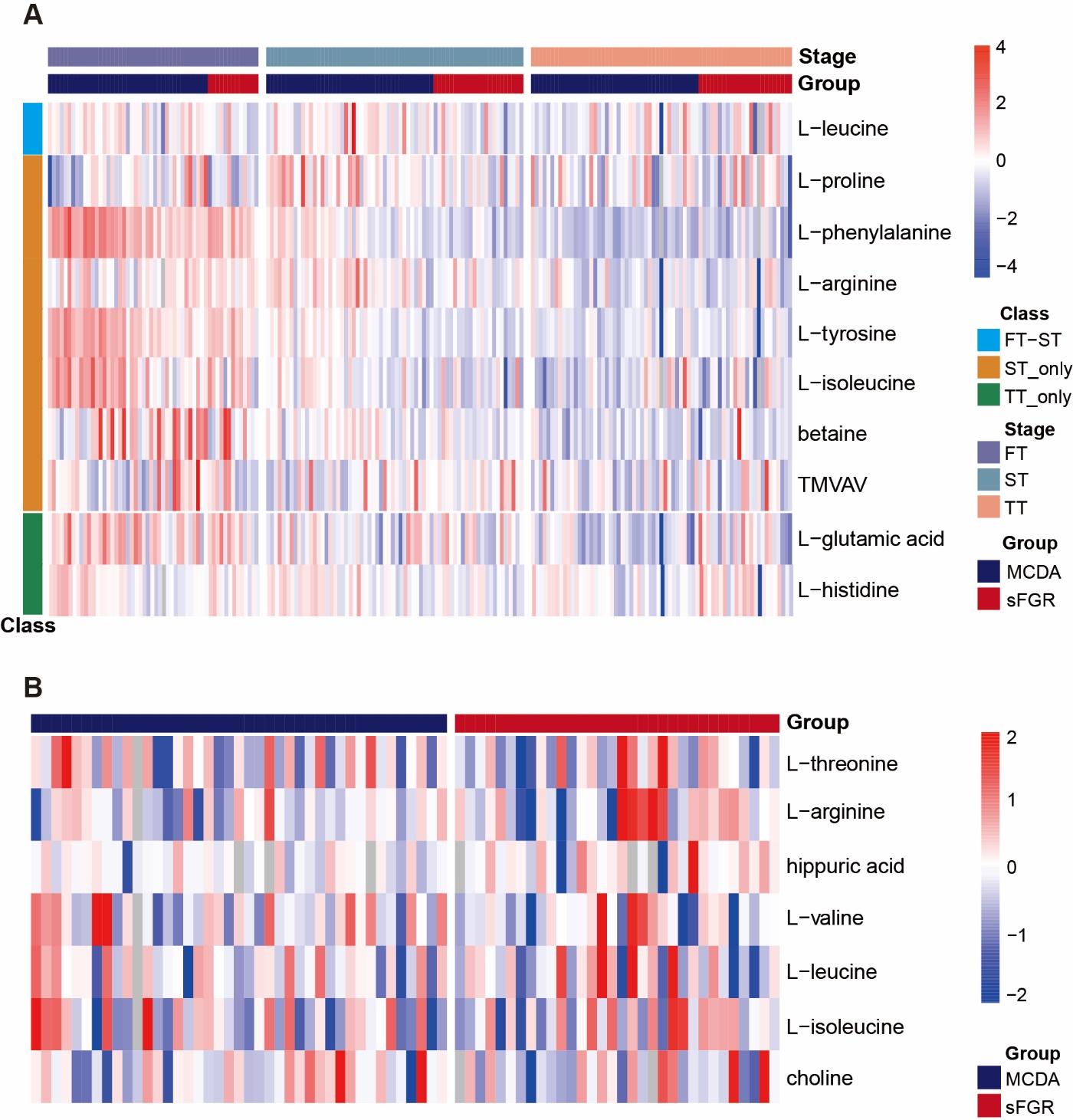
**

**Figure S2 The metabolite concentrations of significantly differential metabolites in maternal plasma and cord plasma.**

A, Heatmap showing the concentrations in maternal plasma in different trimesters for ten significantly differential maternal metabolites identified between MCDA and sFGR groups. Dark blue and dark red bars on the top refer to the MCDA and sFGR group. Purple, dusty blue and pinkish-orange bars on the top refer to the three different trimesters, respectively. Light blue, orange and green bars on the left indicate the classes of significantly differential maternal metabolites, named FT-ST, ST_only and TT_only, respectively. The class of FT-ST means the metabolites both significant in FT and ST between the sFGR and MCDA groups; The class of ST only or TT only means the metabolites only significant ST or TT between the sFGR and MCDA groups; B, Heatmap showing the concentrations in CP for seven significantly differential fetal metabolites identified between MCDA and sFGR. MCDA: normal monochorionic diamniotic twins; sFGR: selective fetal growth restriction; FT: first trimester, ST: second trimester, TT: third trimester; MP: maternal plasma; CP: cord plasma; TMVAV: N, N, N-trimethyl-5-aminovaleric acid;

**
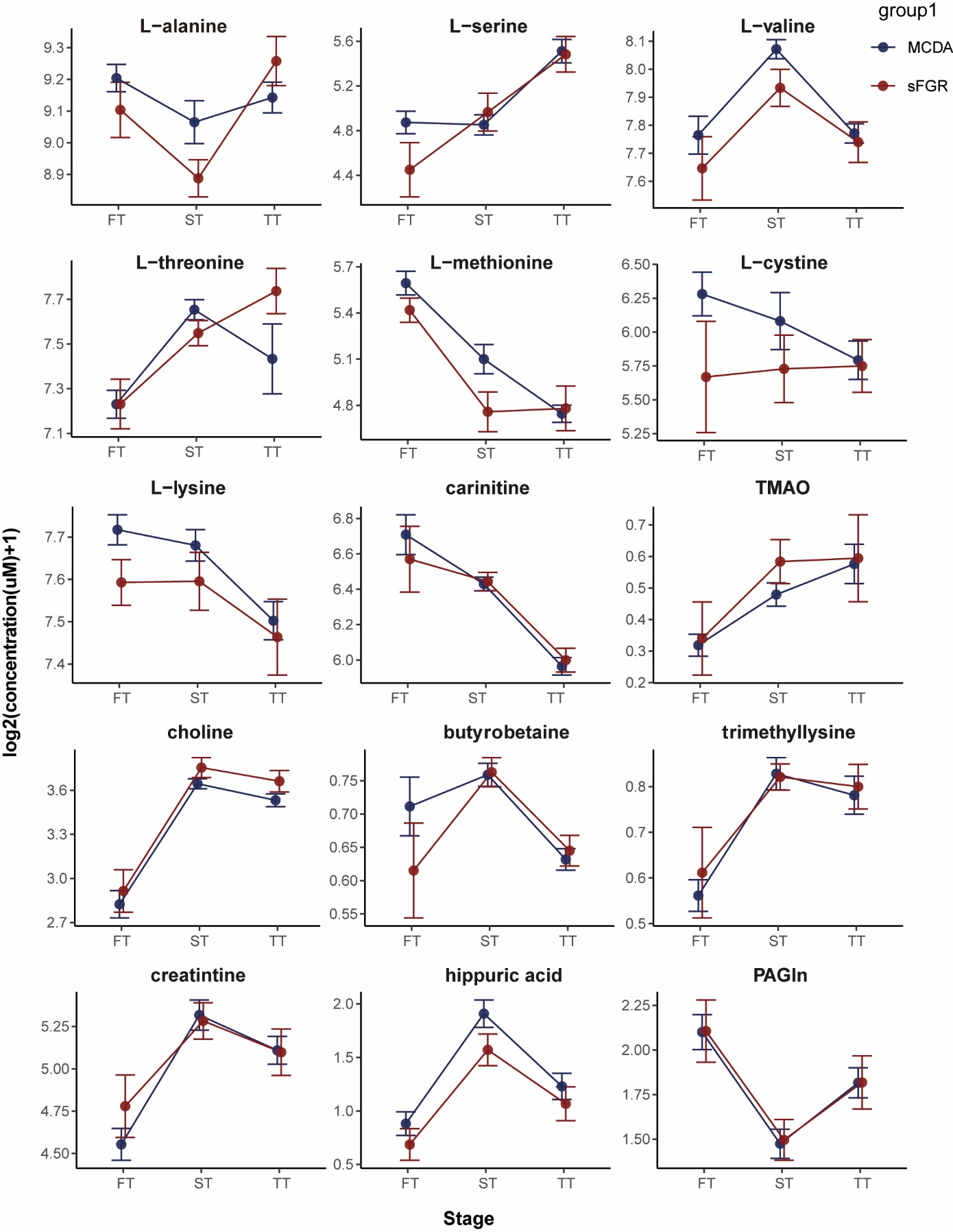
**

**Figure S3 Maternal metabolite levels** **in either the sFGR or MCDA group in different** **trimesters**

Line plot showing the concentrations in maternal plasma during different trimesters for metabolites with no significant difference between the sFGR and MCDA groups. Student’s t-test and Mann‒Whitney U tests were used for parametric or nonparametric comparisons in two groups, respectively. Log2-transformed metabolite concentrations (μM) are presented as the mean ± standard deviation (SD). MCDA: normal monochorionic diamniotic twins; sFGR: selective fetal growth restriction; MP: maternal plasma; FT: first trimester; ST: second trimester; TT: third trimester; TMAO: trimethylamine-N-oxide; PAGln: phenylacetylglutamine;

**
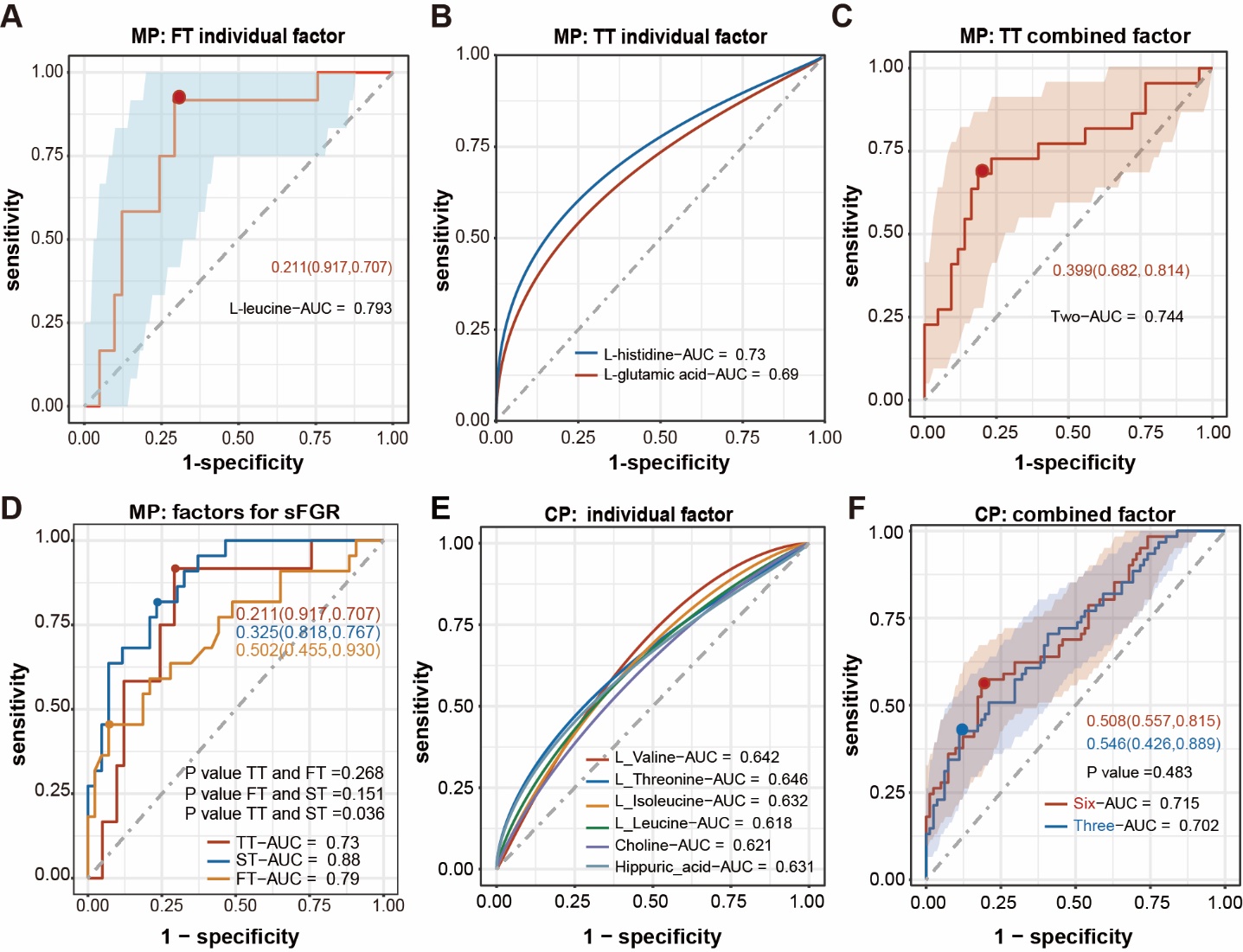
**

**Figure S4 The prediction model of sFGR in** **maternal plasma and cord plasma.**

A, ROC curves of models constructed based on each significantly differential metabolites in the first trimester for the prediction of sFGR; B, ROC curves of models constructed based on each significantly differential metabolites in the third trimester for the prediction of sFGR; C, ROC curve of models constructed based on the combination of significantly differential metabolites for the prediction of sFGR in the third trimester. Two-AUC presents the predictive effectiveness of the optimized combination of two metabolites, including L-histidine and L-glutamic acid; D, Evaluation of the ROC curve of models constructed based on the combination of significantly differential metabolites for the prediction of sFGR in first (L-leucine), second (L-leucine, L-isoleucine, and L-phenylalanine), and third (L-glutamic acid and L-histidine) trimester. The three ROC curves were compared among each other using the DeLong test and *P* values were shown. *P* values in figure D were shown as the significant difference between different trimesters in maternal plasma. E, ROC curves of models constructed based on each significantly differential metabolites in cord plasma for the prediction of sFGR; F, ROC curve of models constructed based on the combination of significantly differential metabolites for the prediction of sFGR in cord plasma. Six-AUC presents the predictive effectiveness of the combination of six metabolites, including L-valine, L- threonine, L-isoleucine, L-leucine, choline, and hippuric acid. The three-AUC presents the predictive effectiveness of the optimized combination of three metabolites, including L-valine, L-isoleucine, and hippuric acid. *P* value in figure F was shown as the significant difference between model A and model B in cord plasma; sFGR: selective fetal growth restriction; MP: maternal plasma; CP: cord plasma; FT: first trimester; ST: second trimester; TT: third trimester; ROC: receiver operating characteristic; AUC: area under curve.

**
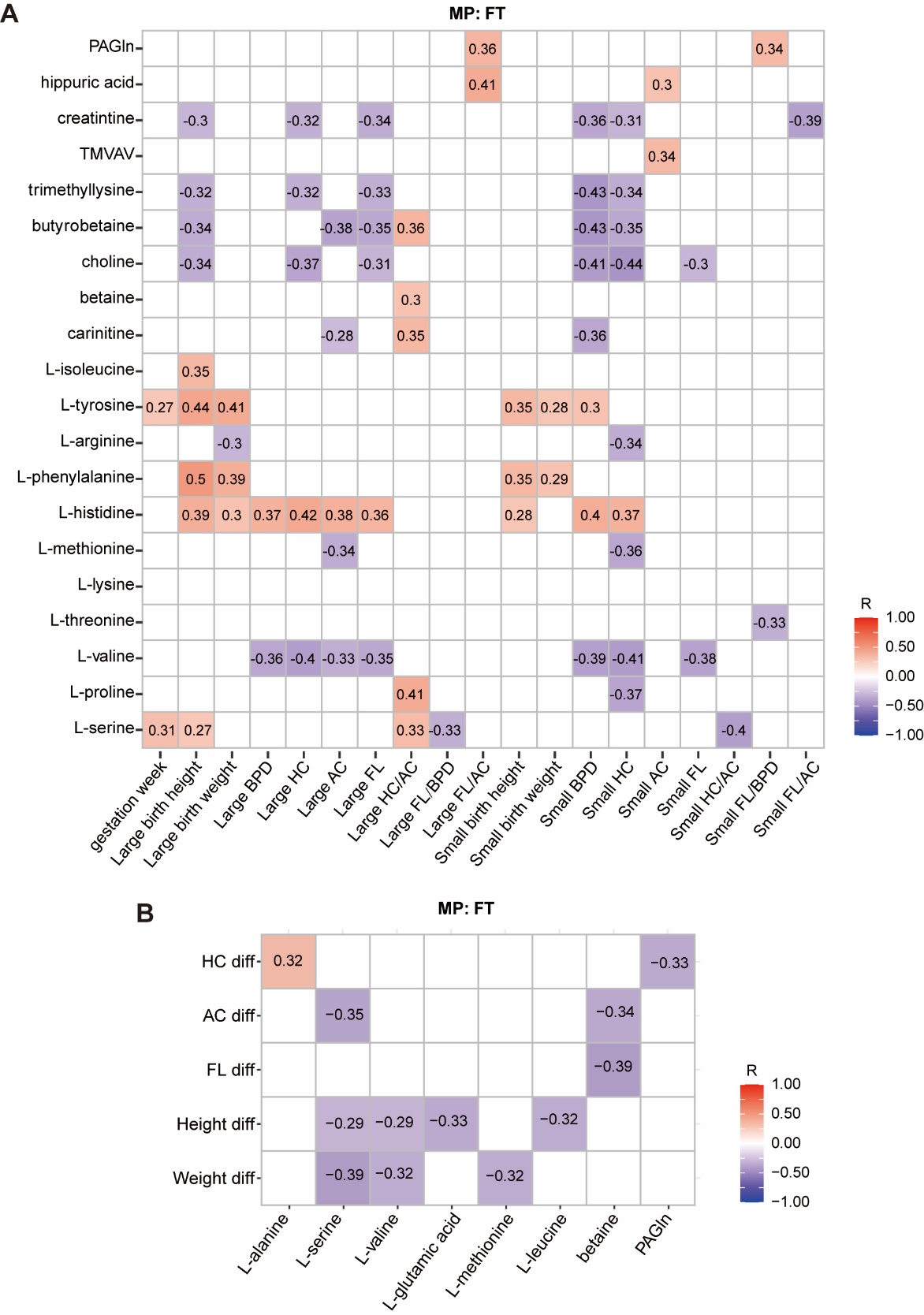
**

**Figure S5** **Correlation between metabolites in** **the first trimester and** **physical development** **parameters.**

A, Correlation between metabolites in the first trimester and physical development parameters between the large and small fetuses. B, Correlation between metabolites in the first trimester and the difference in physical development parameters. The correlation between the metabolites in the first trimester concentration and physical development parameters was examined by Spearman correlation analysis. FT: first trimester; HC: head circumference; AC: abdominal circumference; BPD: biparietal diameter; FL: femur length; S/D: ratio of fetal umbilical artery systolic pressure to diastolic pressure; TMVAV: N, N, N-trimethyl-5-aminovaleric acid; PAGln: phenylacetylglutamine; diff: the difference between the larger and the smaller fetuses;

**
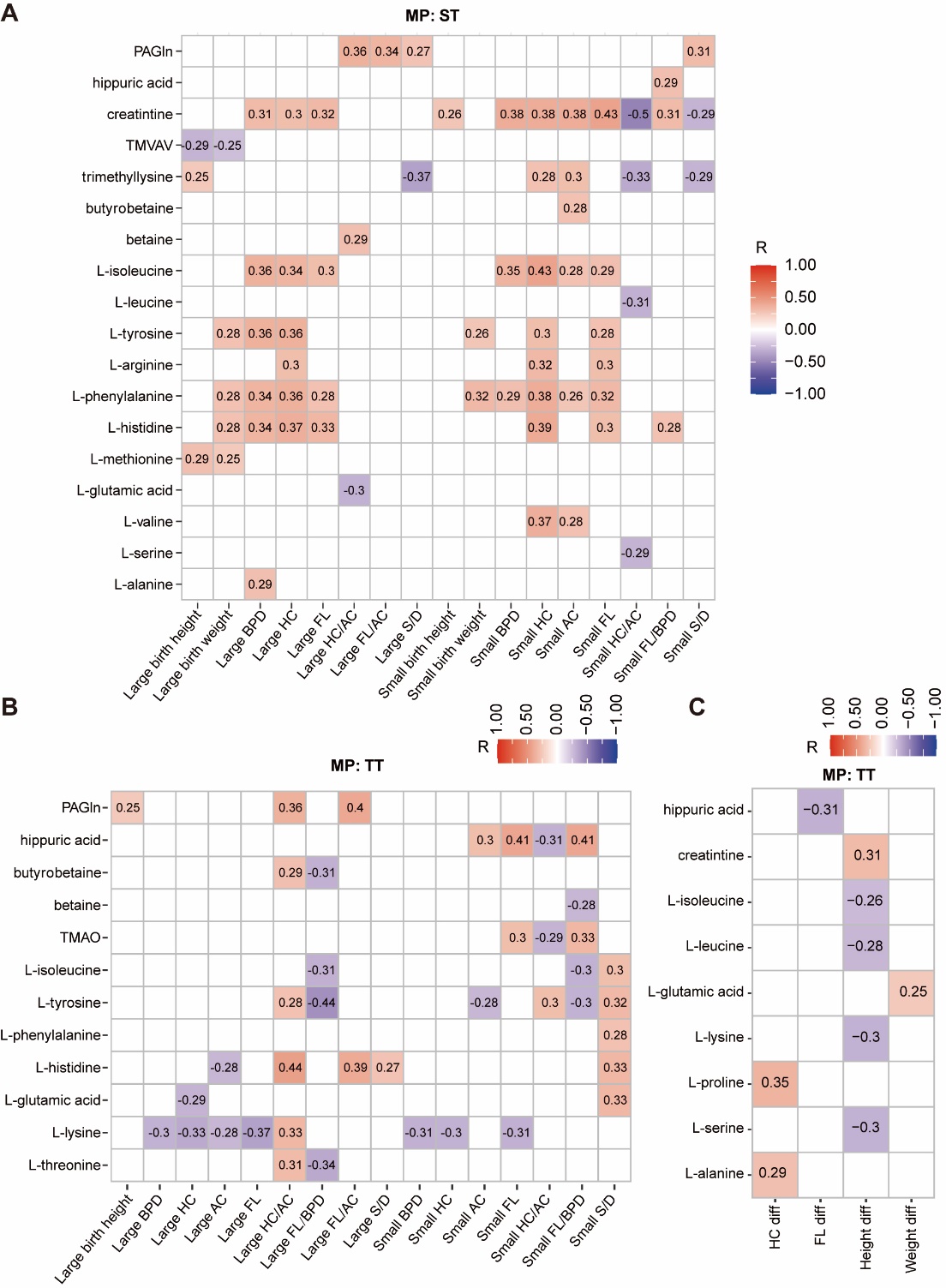
**

**Figure S6 Correlation between metabolites in** **second** **and third trimester, and physical development** **parameters.**

A, Correlation between metabolites in the second trimester and physical development parameters between the large and small fetuses. B, Correlation between metabolites in third trimester and physical development parameters between the large and small fetuses. C, Correlation between metabolites in the third trimester and the difference in physical development parameters. The correlation between the metabolite concentrations and physical development parameters was examined by Spearman correlation analysis; Spearman’s correlation coefficients (R) were used to present the correlations, and only R values with *P*<0.05 were plotted; ST: second trimester; TT: third trimester; MP: maternal plasma; HC: head circumference; AC: abdominal circumference; BPD: biparietal diameter; FL: femur length; S/D: ratio of fetal umbilical artery systolic pressure to diastolic pressure; TMVAV: N, N, N-trimethyl-5-aminovaleric acid; TMAO: trimethylamine-N-oxide; PAGln: phenylacetylglutamine; diff: the difference between the larger and the smaller fetuses;


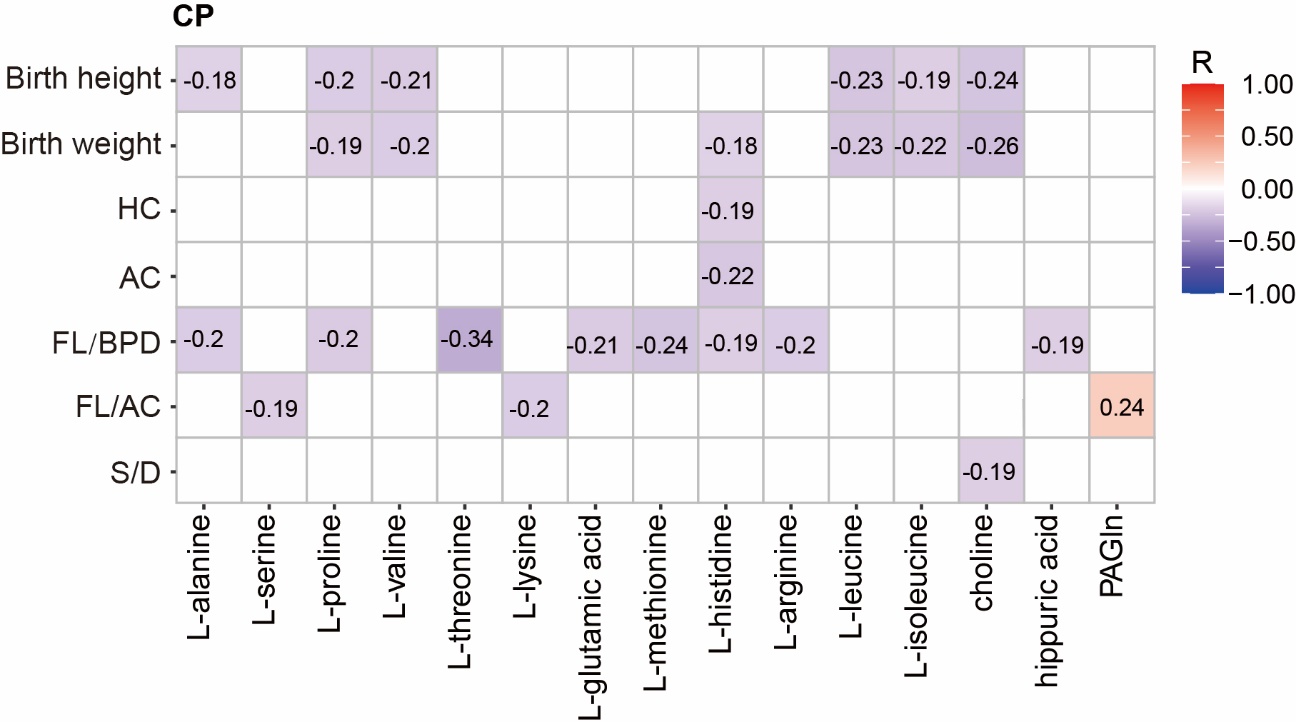


**Figure S7 Correlation between metabolites in cord plasma and** **physical development parameters.**

The correlation between the metabolite concentrations and physical development parameters was examined by Spearman correlation analysis. Spearman’s correlation coefficients (R) were used to present the correlations, and only R values with *P*<0.05 were plotted. HC: head circumference; AC: abdominal circumference; BPD: biparietal diameter; FL: femur length; S/D: ratio of fetal umbilical artery systolic pressure to diastolic pressure;

**
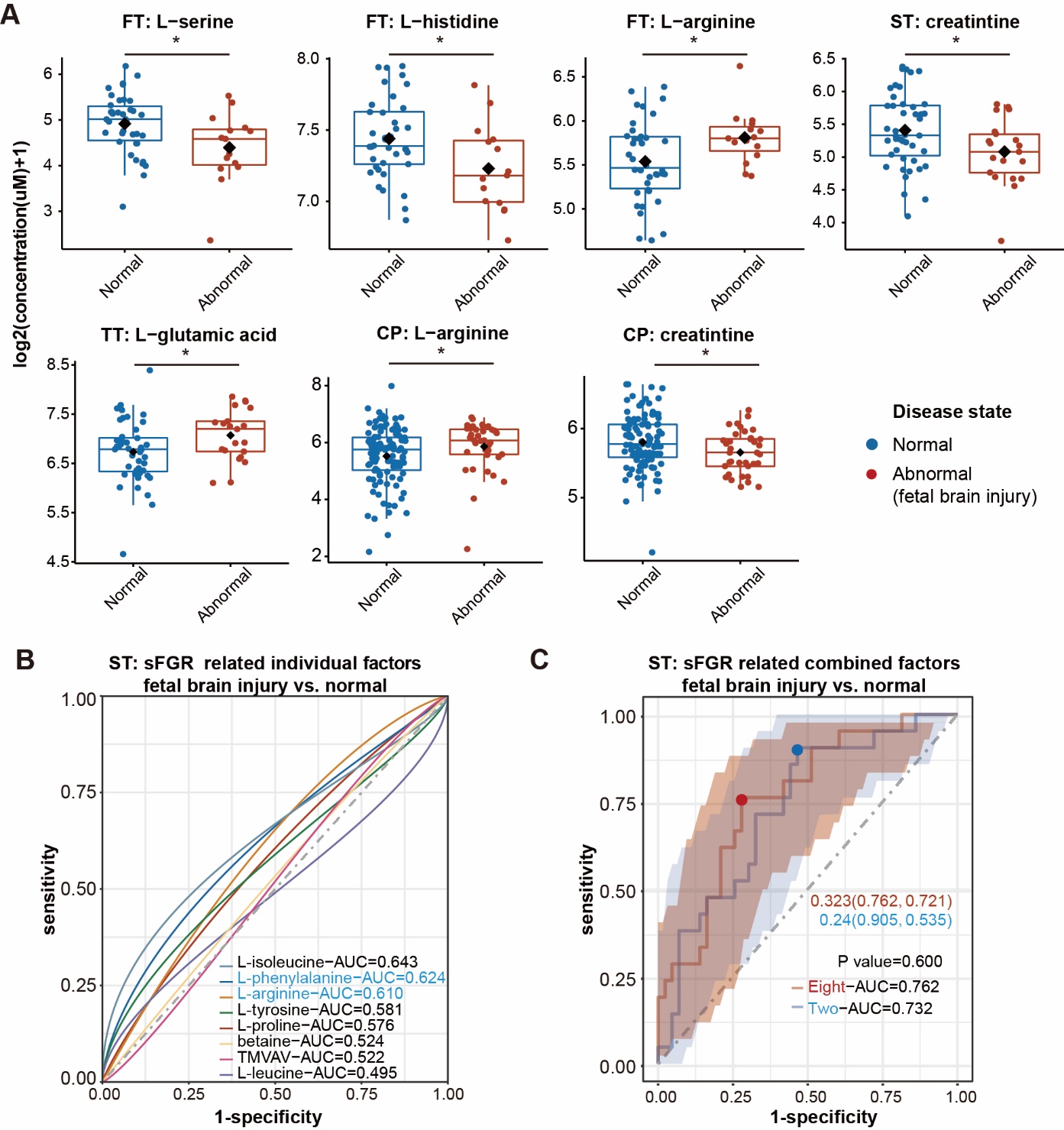
**

**Figure S8 The differential maternal-fetal metabolite levels and the prediction model of fetal brain injury.**

A, Boxplot showing the differential metabolite levels between the normal and abnormal groups in the fetal brain injury group. Data were presented as the median and interquartile range (25%-75%); B, ROC curves of models constructed based on each significantly differential metabolites in the second trimester between the sFGR and MCDA group for the prediction of fetal brain injury. C, ROC curve of models constructed based on the combination of significantly differential metabolites between sFGR and MCDA groups for the prediction of fetal brain injury. Eight-AUC presents the predictive effectiveness of the combination of eight significant metabolites, including L-proline, L-arginine, L-tyrosine, L-leucine, L-isoleucine, L-phenylalanine, betaine, and TMVAV. Two-AUC presents the predictive effectiveness of the optimized combination of two metabolites, including L-arginine and L-phenylalanine; The dots on the broken lines in figure C represent the best cut-off value, and the values in the figure represent the best cut-off value (sensitivity, specificity). The shadow around the linear regression trendline shows the 95% confidence interval (CI). The *P* value in figure C was shown as the significant difference between model A and model B in maternal plasma in the second trimester; sFGR: selective fetal growth restriction; MP: maternal plasma; CP: cord plasma; FT: first trimester; ST: second trimester; TT: third trimester; TMVAV: N, N, N-trimethyl-5-aminovaleric acid; ROC: receiver operating characteristic; AUC: area under curve; * *P*<0.05.

**
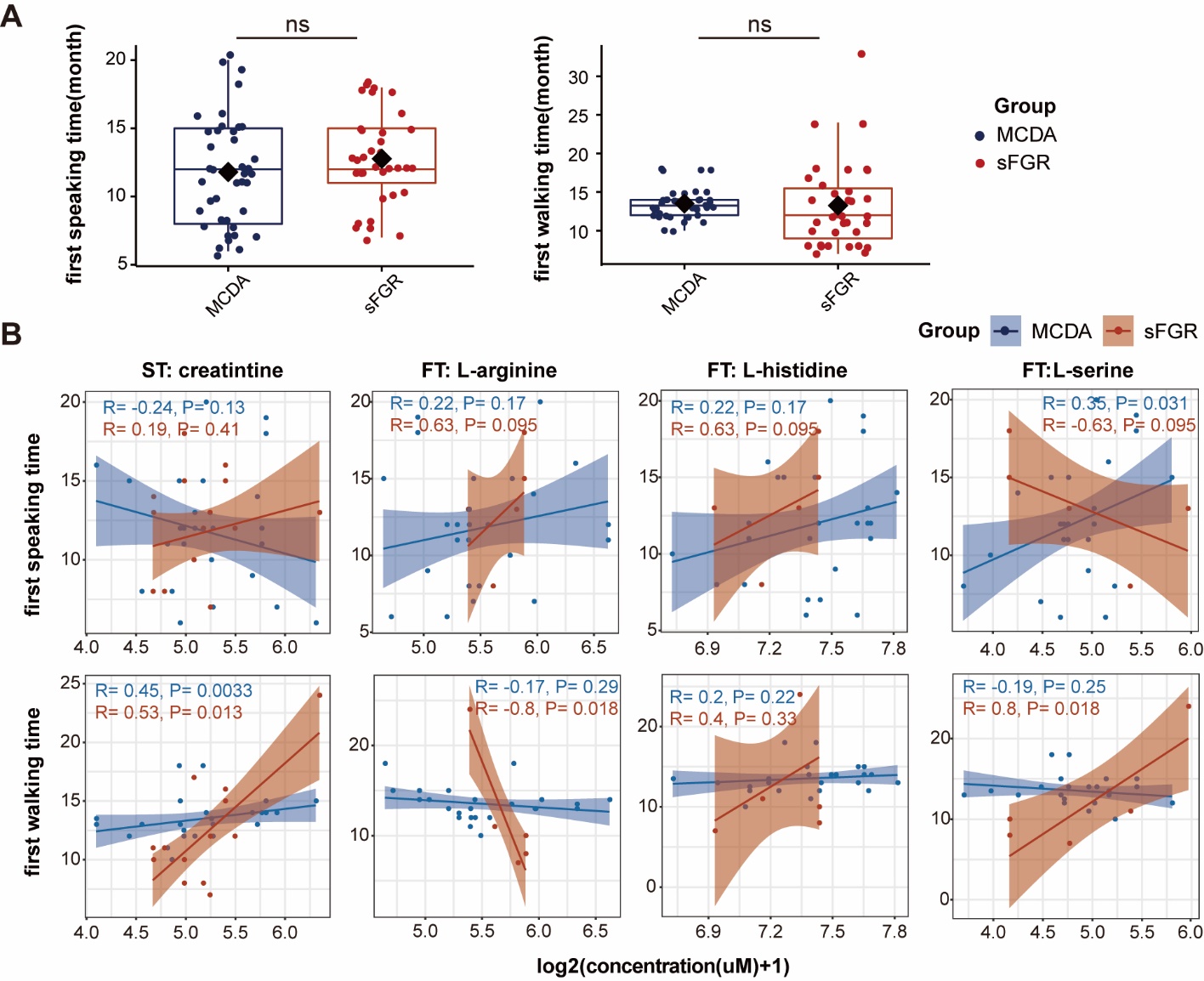
**

**Figure S9 Correlation between maternal metabolites and first speaking and walking times.**

A, Boxplot showing the comparison of the first speaking time, first walking time between the sFGR and MCDA groups; Data were presented as the median and interquartile range (25%-75%); B, The correlation between the four metabolite concentrations and first speaking time and first walking time was examined by Spearman correlation analysis; Spearman’s correlation coefficients (R) were used to present the correlation. The shadow around the linear regression trendline shows the 95% confidence interval (CI); The metabolite level in figure B was determined as Log2-transformed metabolite concentrations (μM). *P*<0.05 was considered statistically significant; ns: nonsignificant.

**
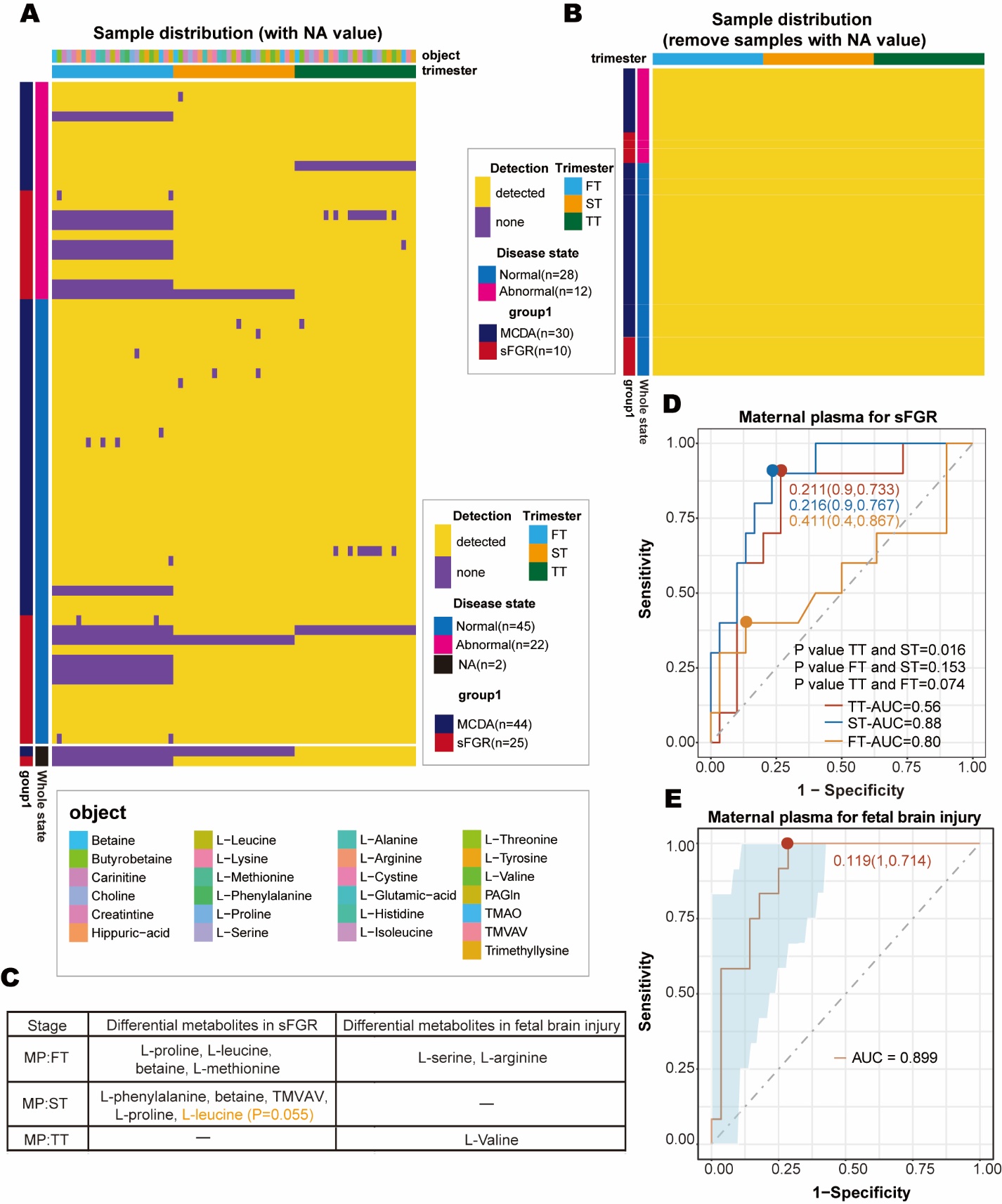
** **Figure S10 Summary for the metabolite profile assessment in sensitivity analysis.**

A and B, Heatmaps showing the detected states for metabolites in maternal plasma among three gestation periods for all the cases covering all cases with missing data (A, n=69) or only cases without any missing data (B, n=40). Purple and yellow boxes in the heatmaps refer to the detected metabolites and the missing data, respectively. The color bars on the top refer to the 25 metabolites. Blue, orange, and green bars on the top refer to three different trimesters, respectively. Dark blue and dark-red bars on the left refer to the MCDA and sFGR groups. Light blue, pink and black bars on the left refer to the fetal brain injury (Abnormal), control (normal), and the cases missing the information of fetal brain injury (NA); C, Summary of sFGR-related or fetal brain injury related intergroup differential metabolites identified in the sensitivity analysis based on the remaining 40 cases referred to in figure B; D, Evaluation of the ROC curves of the models constructed based on the remaining cases in the sensitivity analysis in figure C for the prediction of sFGR in different trimesters. The three ROC curves were compared among each other using the DeLong test and *P* values were shown; E, Evaluation of the ROC curve of models constructed based on the remained 40 cases in sensitivity analysis in figure C for the prediction of fetal brain injury. The AUC presents the predictive effectiveness of the optimized combination of significantly differential metabolites. sFGR: selective fetal growth restriction; MCDA: normal monochorionic diamniotic twins; MP: maternal plasma; FT: first trimester; ST: second trimester; TT: third trimester; ROC: receiver operating characteristic; AUC: area under curve.

**
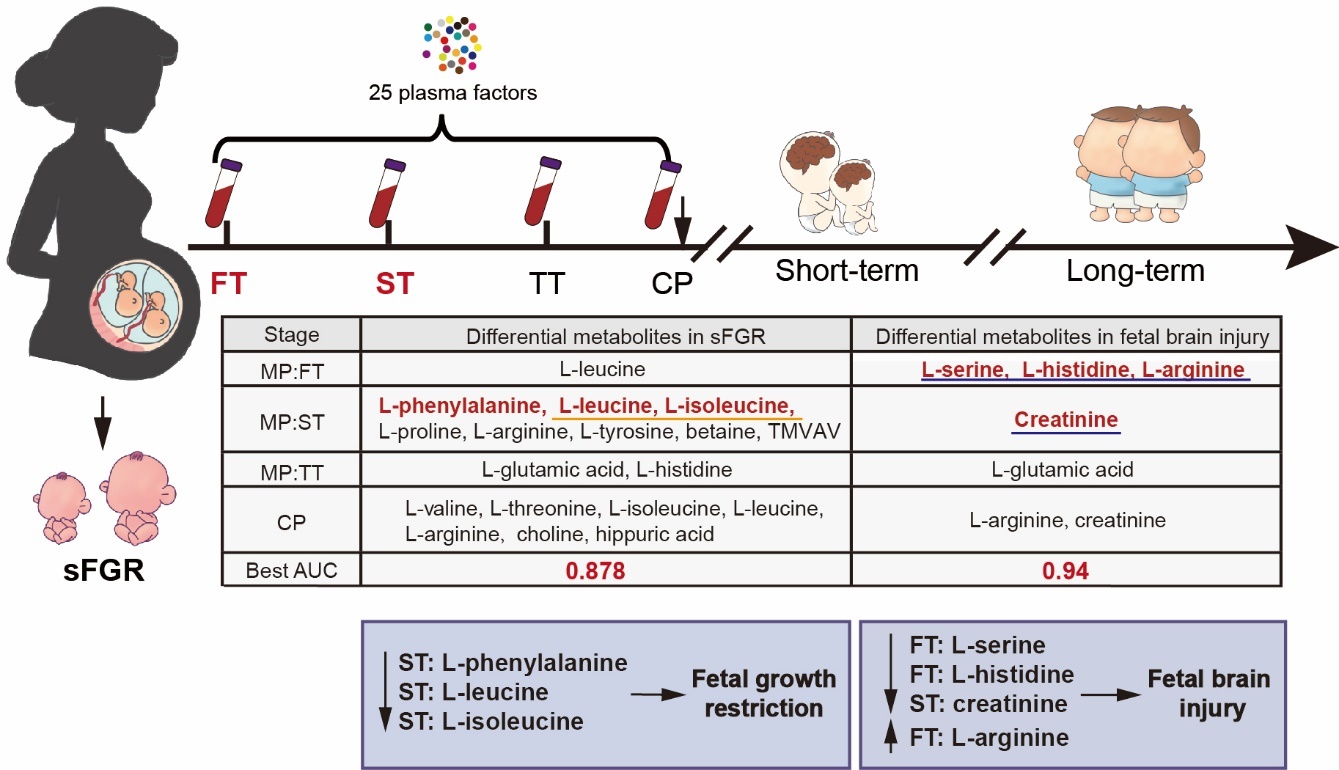
**

**Figure S11 Summary figure of this study.**

Yellow underscores identify metabolites that correlated with long-term age-standardized physical parameters in the prediction model of sFGR. Metabolites that were correlated with the long-term neurocognitive behavioral development in the brain injury prediction model are identified by the blue underscores. Arrows pointing down indicate downregulation of metabolite levels, and arrows pointing up indicate upregulation of metabolite levels; MP: maternal plasma; CP: cord plasma; FT: first trimester, ST: second trimester, TT: third trimester; sFGR: selective fetal growth restriction; TMVAV: N, N, N-trimethyl-5-aminovaleric acid;
